# Serum Carotenoids and Cancer-Related Fatigue: An Analysis of the 2005–2006 National Health and Nutrition Examination Survey

**DOI:** 10.1158/2767-9764.CRC-21-0172

**Published:** 2022-03-31

**Authors:** Amber S. Kleckner, Edwin van Wijngaarden, Todd A. Jusko, Ian R. Kleckner, Po-Ju Lin, Karen M. Mustian, Luke J. Peppone

**Affiliations:** 1 Department of Pain and Translational Symptom Science, University of Maryland School of Nursing, Baltimore, Maryland.; 2 Department of Public Health Sciences, University of Rochester Medical Center, Rochester, New York.; 3 Division of Supportive Care in Cancer, Department of Surgery, University of Rochester Medical Center, Rochester, New York.

## Abstract

Cancer-related fatigue is a prevalent, debilitating condition, and preliminary evidence suggests a relationship between higher diet quality and lower fatigue. Serum-based carotenoids, Vitamin A, and Vitamin E are biomarkers of fruit and vegetable intake and therefore diet quality. To further elucidate the link between diet quality and cancer-related fatigue, associations were assessed between these serum-based nutrients and fatigue among American adults with special attention to cancer history. Data were analyzed from the United States 2005–2006 National Health and Nutrition Examination Survey dataset. Ten carotenoids, vitamin A, vitamin E, and γ-tocopherol were measured from fasting blood samples and fatigue was patient-reported. Associations between carotenoid concentration and fatigue were estimated using ordinal logistic regression models. Adjusted models included a diagnosis of cancer (with the exception on nonmelanoma skin cancer, yes/no), age, body mass index, race/ethnicity, education, and exercise habits as covariates, and additional models included a cancer × nutrient interaction. Of 4,091 participants, 272 (8.0%) reported a history of cancer. Greater fatigue was associated with lower serum *trans*-lycopene, retinyl palmitate, and retinyl stearate (all *P* < 0.05) in separate models adjusting for potential confounders. For these nutrients, a one-SD increase in nutrient was associated with a 6.8%–9.9% lower risk of greater fatigue. Among cancer survivors only (*n* = 272), statistically significant associations were not observed between any of the nutrients and fatigue. In conclusion, greater serum concentrations of carotenoid biomarkers were associated with less fatigue. These results support further exploration into relationships between carotenoid intake, diet quality, and persistent fatigue.

**Significance:**

Cancer-related fatigue often persists for years into survivorship, reduces quality of life, and prevents people from returning to their lives before cancer. Interventions to address cancer-related fatigue are much needed. Herein, serum carotenoids were associated with lower fatigue, thereby supporting further development of nutritional interventions to address fatigue in survivorship.

## Introduction

Cancer-related fatigue affects at least 30%–90% of patients with cancer, depending on the type of cancer and the treatment (surgery, chemotherapy, and/or radiation; refs. 1–6). Cancer-related fatigue is one of the most prevalent and distressing side effects of the cancer experience, and approximately one-third of cancer survivors experience chronic fatigue for years after treatment (5, 6). It is not relieved by sleep or rest, and its severity can impair the ability to perform activities of daily living and reduce quality of life (6, 7). While acute fatigue during treatment is often attributed to treatment-related iron-deficient anemia, inflammation, or other mechanisms, persistent fatigue in survivorship is more elusive (8). Highlighting its severity, several prospective studies have shown an association between cancer-related fatigue with increased mortality (7).

Nutritional status can modulate cancer-related fatigue during treatment and into survivorship. Up to 80% of patients with cancer have clinical malnutrition (regardless of weight status), and poor nutritional status—often defined by recent weight loss—is associated with greater cancer-related fatigue (9, 10). A study among 770 female breast cancer survivors demonstrated an association between less fatigue and higher diet quality—defined using the Healthy Eating Index-2010, which rewards fruit, vegetables, beans, whole grains, dairy, protein, and healthy fats and penalizes high refined grains, sodium, added sugar, and saturated fat (11). It is unknown whether poor-/malnutrition contributes to reversible or irreversible metabolic or physiologic damage during cancer treatment, although early nutritional intervention and dietary counseling can significantly improve nutritional status and quality of life during treatment (9, 12–14) and improve fatigue in survivorship (15). For example, in a randomized controlled trial among breast cancer survivors, a 3-month diet high in fruit, vegetables, whole grains, and omega-3 fatty acids reduced cancer-related fatigue compared with a general health curriculum (15). Moreover, diet and obesity are closely related, and obesity is a risk factor for exacerbated side effects in survivorship, including cancer-related fatigue (16, 17).

Dietary assessment by means of patient-report (e.g., food frequency questionnaires, food diaries, food recall) tends to have a high error rate due to recall bias, difficulty in estimating serving sizes, social desirability bias, and other factors (18). Blood-based biomarkers have emerged as objective surrogates for specific foods and nutrients and may serve as a proxy for diet quality, specifically, serum carotenoid concentrations (18, 19).

Serum carotenoids are surrogates for diet quality because they are plentiful in many fruits and vegetables (19, 20). Carotenoids are high in fruits and vegetables that have a yellow, red, or orange hue, including carrots, cantaloupe, sweet potatoes, tomatoes, and apricots, as well as leafy green vegetables in which the chlorophyll masks the carotenoid pigments, including kale, broccoli, and collard greens. Carotenoids are relatively specific to fruits and vegetables, with the exception of egg yolks, which are one of the only animal products that contain carotenoids. Consequently, carotenoids are established as reliable biomarkers of fruit and vegetable intake (21). Carotenoids include lycopene, β-carotene, lutein, zeaxanthin, and others, and some are provitamins that are converted into vitamin A in the body. Because people tend to eat a fairly consistent diet in adulthood (22, 23), carotenoid biomarkers from fasting blood samples are considered reliable for epidemiology studies, with less than 8% variation in blood concentrations of lycopene, β-cryptoxanthin, and lutein over four years (24).

Several studies have suggested that serum carotenoids may correlate with cancer-related fatigue or fatigue in general. For example, an inverse association was observed between carotenoid intake and fatigue among 40 adult cancer survivors in a cross-sectional study in the United States (25). In addition, in a study exploring the effects of nutritional biomarkers on sleep factors in the 2005–2006 National Health and Nutrition Examination Survey (NHANES) dataset, an inverse association was observed between carotenoids [retinyl esters and lutein + zeaxanthin (combined predictor)] and poor sleep-related daytime dysfunction (not necessarily cancer-related fatigue) adjusting for history of cancer among other comorbidities (26).

However, there have been no studies to our knowledge assessing serum carotenoids specifically on fatigue in the context of cancer survivorship. Thus, we evaluated the associations between serum carotenoid concentrations and fatigue, with special attention to cancer survivors compared with individuals without a prior cancer diagnosis. These data are hypothesis generating, yet shed light on associations between serum nutrient biomarkers and cancer-related fatigue and can help guide intervention development (nutritional supplements, meal replacements, and dietary interventions) to alleviate cancer-related fatigue in survivorship.

## Materials and Methods

### Study Population

NHANES is an initiative by the U.S. Department of Health and Human Services, Centers for Disease Control and Prevention, and National Center for Health Statistics to collect objective and patient-reported data regarding the health and nutritional status of nationally representative civilian noninstitutionalized children and adults living in the United States. It is a continuous program, and the 2005–2006 NHANES oversampled adolescents, older Americans, African Americans, and Mexican Americans to address specific needs of these underrepresented populations. NHANES is conducted in accordance with ethical guidelines—the protocol was approved by the National Center for Health Statistics Research Ethics Review Board and all participants provided written informed consent (27, 28). Trained NHANES professionals administered cross-sectional surveys in a multistage probability cluster sampling design using at-home interviews and health examinations using a mobile examination center. The details of recruitment and data collection have been previously described (27, 28). The 2005–2006 NHANES dataset was selected for this analysis because it included serum carotenoid measures, cancer status, and a question on daytime fatigue.

The University of Rochester Research Subjects Review Board reviewed the protocol for this analysis and declared it exempt (STUDY00006373). Participants were excluded from this analysis if they had missing data for the question regarding a history of a cancer diagnosis (yes/no; *n* = 565), the Depression Screener Questionnaire (the source of the fatigue outcome variable; DPQ; *n* = 503), and/or carotenoid laboratory values (*n* = 391). Because nonmelanoma skin cancer is usually treated with surgery under a local anesthetic and not chemotherapy or radiation, we included participants with a single cancer diagnosis of nonmelanoma skin cancer in the group without a recent cancer diagnosis (*n* = 57). We then performed a sensitivity analysis with these participants in the group with a recent cancer diagnosis; estimates for the association between nutrient and fatigue changed less than 10% in all models except those for *cis*- and *trans*-beta carotene and vitamin A, all of which had estimates <0.002 units.

### Fatigue Measurement

The DPQ, also referred to as the Patient Health Questionnaire-9, is a nine-item questionnaire that is based on the Diagnostic and Statistical Manual of Mental Disorders, Fourth Edition (DSM-IV) and has been validated in the general population (29, 30). Daytime fatigue was assessed via one question from the DPQ (DPQ040): “Over the last 2 weeks, how often have you been bothered by the following problem[s]: feeling tired or having little energy?” Response options were “not at all” (0 or none), “several days” (1 or light), “more than half the days” (2 or moderate), and “nearly every day” (3 or severe). While NHANES did not include a question specific to cancer-related fatigue, a single-item question such as this one is often used to assess patient-reported fatigue (31, 32).

### Measurement of Serum Carotenoids

Blood was collected via venipuncture after an overnight (>9 hour) fast by a trained phlebotomist into a red-top or royal blue-top Vacutainer blood tube (33). Serum was harvested using standard procedures and stored at −70°C until analysis (up to 2 years). Carotenoids—total (*cis*- and *trans*-) lycopene, α-carotene, *trans*-β-carotene, *cis*-β-carotene, β-cryptoxanthin, lutein + zeaxanthin, *trans*-lycopene, retinyl palmitate, retinyl stearate; vitamin A; and vitamin E were quantified at one of 28 laboratories across the United States using high-performance liquid chromatography (HPLC) with photodiode array detection; methods are detailed in the NHANES documentation (28).

### Other Covariates

The fully adjusted models controlled for potential confounders as supported by the literature including age (34), body mass index (BMI; refs. 16, 34, 35), race/ethnicity (35–37), education (38), and exercise habits (4).

### Statistical Analyses

All statistical analyses were performed in SAS, version 9.4 (SAS Institute). Descriptive statistics for demographics and clinical characteristics (means, proportions) were calculated for the overall population and in relation to cancer history (yes/no). Age was considered a continuous variable and BMI was coded as a categorical variable with traditional cut-off points (underweight, <18.5 kg/m^2^; normal weight, 18.5–<25 kg/m^2^; overweight, 25–<30 kg/m^2^; or obese, ≥30 kg/m^2^; ref. 16). Race/ethnicity was categorized into Mexican American, Other Hispanic, non-Hispanic White, non-Hispanic Black, and other non-Hispanic race including multiracial. Education was categorized into less than a high school education, less than a college degree, or at least a four-year college degree. Habitual exercise was estimated using patient-reported metabolic equivalents (MET) hours per week and was treated as a continuous variable. To adjust for the complex sampling design, oversampling of select populations, and nonresponse, the data were weighted so that our results represent the total U.S. population. Specifically, appropriate adjustments were applied for strata (sdmvstra), cluster (sdmvpsu), and Mobile Examination Center weight (wtmec2yr). Using ordinal logistic regression (PROC SURVEYFREQ) and appropriate sample weights, we assessed the relationship between fatigue [question DPQ040 as an ordinal variable (0, 1, 2, 3)] and each of the nutrient concentrations in independent models. We performed a crude analysis as well as one adjusting for cancer diagnosis (excluding nonmelanoma skin cancer, yes or no), age, BMI, race/ethnicity, education, and exercise. To assess effect modification between serum carotenoid concentration and a history of a cancer diagnosis, an interaction term was included in the adjusted models and stratum-specific estimates are reported for those with and without a history of cancer (excluding nonmelanoma skin cancer). *P* < 0.05 was considered statistically significant.

### Data Availability

The data analyzed in this study are publicly available from NHANES at https://www.cdc.gov/nchs/nhanes/.

## Results

A total of 4,091 participants were included in this analysis, of which 272 (8.0%) had a history of a cancer diagnosis (with the exception of nonmelanoma skin cancer; Table 1). Individuals with a history of cancer were, on average, 15.5 years older than those without a cancer diagnosis (*P* < 0.001) and there was a slightly greater proportion of females among those with a cancer diagnosis (*P* < 0.001). Interestingly, there were notable differences in the distribution of races and ethnicities among those with and without a history of cancer—9.9% of non-Hispanic Whites had had a cancer diagnosis while only 2.1% of Mexican Americans, 4.1% of other Hispanic Americans, 4.2% of Black Americans, and 2.7% of those of mixed race or other races had had a cancer diagnosis (*P* < 0.001). In line with typically reported statistics (39), approximately two-thirds of all participants were overweight or obese, with no significant between-group differences (*P* = 0.85). Among those with a history of cancer compared to those without a history of cancer, serum concentrations of some carotenoids and vitamins tended to be higher (e.g., *trans*-β-carotene, vitamin A, both *P* < 0.01), some tended to be lower (e.g., total lycopene, γ-tocopherol, both *P* < 0.01), and some were similar (e.g., lutein and zeaxanthin, *P* = 0.56). Those with a history of cancer reported a greater daytime fatigue score than those without a history of cancer (scale from 0–4, mean ± SE, 0.87 ± 0.08 vs. 0.69 ± 0.02, two-sided *t* test *P* = 0.03).

**TABLE 1:** Demographics and clinical characteristics.^a^

	Total (*n* = 4,091)	With a history of a cancer diagnosis^b^ (*n* = 272)	No history of cancer (*n* = 3,819)	*P* ^c^
Age (years)	46.7 ± 0.7	61.1 ± 1.2	45.6 ± 0.7	<0.001
Gender				<0.001
Male	1,986 (48.4%, 48.5%)	109 (40.1%, 31.1%)	1,872 (49.0%, 49.7%)	
Female	2,110 (51.6%, 51.5%)	187 (68.8%, 68.9%)	1,947 (51.0%, 50.3%)	
Race/ethnicity				<0.001
Hispanic, not Mexican American	123 (3.0%, 3.2%)	5 (1.8%, 1.8%)	118 (3.1%, 3.3%)	
Mexican American	826 (20.2%, 7.8%)	17 (6.3%, 2.2%)	809 (21.2%, 8.2%)	
Non-Hispanic Black	880 (21.5%, 10.5%)	37 (13.6%, 5.1%)	843 (22.1%, 10.9%)	
Non-Hispanic White	2,116 (51.7%, 73.8%)	209 (76.8%, 88.1%)	1,907 (49.9%, 72.8%)	
Other non-Hispanic race, including multi-racial	146 (3.6%, 4.7%)	4 (1.5%, 2.7%)	142 (3.7%, 4.8%)	
Education				0.504
Less than 9th grade	481 (11.8%, 6.0%)	37 (13.6%, 8.8%)	444 (11.6%, 5.8%)	
9–12 grades (no diploma)	622 (15.2%, 10.9%)	33 (12.1%, 8.8%)	589 (15.4%, 11.0%)	
High school graduate/GED or equivalent	966 (23.6%, 24.6%)	71 (26.1%, 27.6%)	895 (23.4%, 24.4%)	
Some college or AA degree	1,178 (28.8%, 31.7%)	67 (24.6%, 27.4%)	1,111 (29.1%, 32.0%)	
College graduate or above	842 (20.6%, 26.8%)	64 (23.5%, 27.4%)	778 (20.4%, 26.7%)	
Don't know	2 (0.05%, 0.04%)	0 (0%, 0%)	2 (0.1%, 0.0%)	
Body mass index (BMI)				0.851
Underweight, BMI < 18.5 kg/m^2^	69 (1.7%, 1.8%)	6 (2.2%, 2.5%)	63 (1.6%, 1.7%)	
Normal weight, 18.5 ≤ BMI < 25 kg/m^2^	1,146 (28.0%, 30.8%)	81 (29.8%, 31.8%)	1,064 (27.9%, 30.8%)	
Overweight, 25 ≤ BMI < 30 kg/m^2^	1,405 (34.3%, 32.8%)	96 (35.3%, 33.3%)	1,307 (34.2%, 32.7%)	
Obese, BMI ≥ 30 kg/m^2^	1,436 (35.1%, 34.6%)	85 (31.3%, 32.4%)	1,349 (35.3%, 34.8%)	
Missing	40 (1.0%, NA)	4 (1.5%, NA)	36 (0.9%, NA)	
Years since cancer diagnosis	NA	10.9 ± 1.1	NA	NA
Exercise habits (Metabolic equivalents (METs), hours per week)	18.2 ± 0.8	15.9 ± 3.2	18.4 ± 0.8	0.440
Serum carotenoid concentrations (μg/dl)				
α-Carotene	4.8 ± 0.2	5.5 ± 0.5	4.8 ± 0.2	0.132
*trans*-β-Carotene	19.3 ± 0.6	25.1 ± 3.1	18.9 ± 0.6	0.048
*cis*-β-Carotene	1.20 ± 0.04	1.6 ± 0.2	1.18 ± 0.04	0.044
β-Cryptoxanthin	9.8 ± 0.3	8.5 ± 0.4	9.9 ± 0.3	0.002
γ-Tocopherol	221.0 ± 4.6	196.7 ± 7.2	222.7 ± 4.6	<0.001
Lutein and zeaxanthin	16.5 ± 0.3	16.9 ± 0.7	16.5 ± 0.3	0.557
*trans*-Lycopene	24.2 ± 0.3	21.2 ± 0.9	24.4 ± 0.3	0.005
Retinyl palmitate	2.62 ± 0.04	2.7 ± 0.2	2.61 ± 0.04	0.560
Retinyl stearate	0.65 ± 0.01	0.69 ± 0.06	0.65 ± 0.01	0.402
Vitamin A	60.7 ± 0.5	65.3 ± 1.2	60.4 ± 0.5	<0.001
Vitamin E	1,263 ± 14	1,467 ± 36	1,248 ± 14	<0.001
Total (*cis*- and *trans*-) lycopene	44.9 ± 0.4	39.7 ± 1.7	45.3 ± 0.4	0.006
Fatigue^d^	0.70 ± 0.02	0.87 ± 0.08	0.69 ± 0.02	0.033

Abbreviations: AA, Associate of Arts; GED, general education development; NA, not applicable

^a^Mean ± SE of the mean for continuous variables with appropriate sampling weights or *N* (crude %, weighted %) for categorical variables.

^b^With the exception of non-melanoma skin cancer.

^c^ *P* value indicates differences between those with and without a history of cancer; it was derived from a *t* test for continuous variables and a Wald *χ*^2^ test for categorical variables.

^d^Fatigue was measured using a single question from the Depression Screener Questionnaire: DHQ040, range 0–3, higher score is worse fatigue.

**FIGURE 1:**
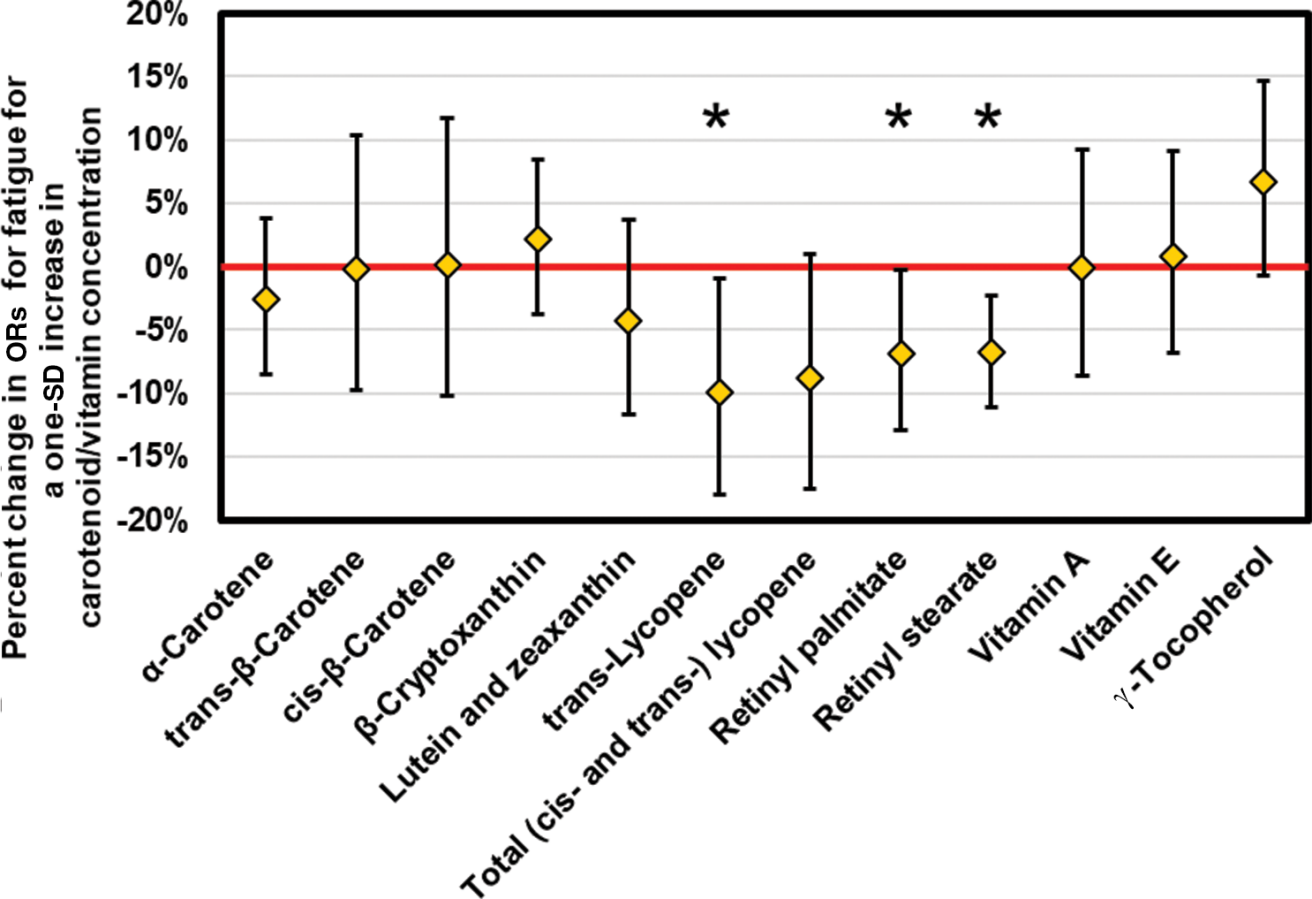
The percent change in odds ratios for experiencing one-point worse fatigue on the Depression Screener Questionnaire fatigue question (DPQ040) for an increase in one SD of serum carotenoid/vitamin concentration (*n* = 4,091). These models were adjusted for age, race/ethnicity, body mass index, education, exercise habits, and history of cancer. *, *P* < 0.05.

We modeled the associations between nutrient concentrations and fatigue. Table 2 and Supplementary Table S1 illustrate the results of crude and fully adjusted models for each of the measured carotenoids, vitamin A, and vitamin E. In models adjusted for age, BMI, race/ethnicity, education, exercise habits, and cancer history, higher serum carotenoid concentrations were associated with lower risk for fatigue for *trans*-lycopene, retinyl palmitate, and retinyl stearate (*P* < 0.05). These ORs indicate that, assuming a linear association between nutrients and fatigue, a one-SD increase in *trans*-lycopene (11.4 μg/dL) is associated with a 9.9% lower risk of more severe fatigue (95% CI, 1.0%-1-8.0%; Fig. 1).

**TABLE 2:** Percent change in ORs for having worse fatigue for a one-SD increase in carotenoid/vitamin concentration.

		Unadjusted model				Adjusted model			
Variables	SD for the distribution for the whole cohort	Percent change in OR	Lower 95% CI	Upper 95% CI	*P*	Percent change in OR	Lower 95% CI	Upper 95% CI	*P*
α-Carotene	6.5	−6.4%	−12.1%	−0.4%	0.039^a^	−2.5%	−8.5%	3.8%	0.401
*trans*-β-Carotene	22.2	−5.1%	−13.3%	3.9%	0.238	−0.2%	−9.7%	10.4%	0.971
*cis*-β-Carotene	1.3	−5.0%	−13.5%	4.4%	0.263	0.1%	−10.3%	11.8%	0.978
β-Cryptoxanthin	9.8	−3.7%	−10.7%	3.8%	0.303	2.1%	−3.8%	8.4%	0.463
γ-Tocopherol	124.2	11.0%	2.6%	20.2%	0.013^a^	6.7%	−0.7%	14.6%	0.076
Lutein and zeaxanthin	9.8	−8.4%	−14.2%	−2.2%	0.012^a^	−4.3%	−11.7%	3.7%	0.257
*trans*-Lycopene	11.4	−9.6%	−16.4%	−2.3%	0.015^a^	−9.9%	−18.0%	−1.0%	0.033^a^
Retinyl palmitate	1.9	−9.6%	−14.8%	−4.0%	0.003^a^	−6.8%	−13.0%	−0.2%	0.043^a^
Retinyl Stearate	0.48	−8.9%	−14.1%	−3.4%	0.004^a^	−6.8%	−11.1%	−2.3%	0.006^a^
Vitamin A	18.1	−4.2%	−12.0%	4.3%	0.300	−0.1%	−8.6%	9.2%	0.986
Vitamin E	509	−3.5%	−9.7%	3.4%	0.300	0.8%	−6.9%	9.1%	0.828
Total (*cis*- and *trans*-) lycopene	21	−8.8%	−16.2%	−0.8%	0.033^a^	−8.8%	−17.6%	0.9%	0.072

NOTE: Adjusted models contained the following covariates: age, race/ethnicity, body mass index, education, exercise habits, and history of cancer (except non-melanoma skin cancer, *n* = 4,091).

^a^ *P* < 0.05.

With regard to covariates (Supplementary Table S1), a cancer diagnosis tended to be associated with greater odds of fatigue (ORs range 1.36–1.48, *P* < 0.05 for all nutrients). Habitual exercise was associated with a lower odds of fatigue (OR = 0.99, *P* < 0.01 for all nutrients). In addition, obesity (BMI ≥ 30 kg/m^2^) tended to be associated with greater patient-reported fatigue for some nutrients (e.g., *trans*-β-carotene, OR, 1.29; 95% CI, 1.01–1.64; *trans*-lycopene, OR, 1.29; 95% CI, 1.01–1.66). In regard to race/ethnicity, Mexican ethnicity tended to be associated with less fatigue than non-Hispanic Whites (ORs range, 0.70–0.73, *P* < 0.04 for all nutrients).

To explore whether nutrient status and the cancer experience synergistically contribute to fatigue, we assessed whether there was effect modification between nutrient concentration and cancer. A statistically significant nutrient–cancer interaction was revealed for lutein and zeaxanthin (OR, 0.97; 95% CI, 0.94–1.00; *P* = 0.04); ORs for all nutrients ranged from 0.81–1.01 (Supplementary Tables S2 and S3). Looking at cancer survivors only, we did not see statistically significant associations between any of the nutrients and fatigue (Table 3; Supplementary Table S4). Low education was significantly associated with greater fatigue among cancer survivors (ORs range, 1.88–2.22 for having less than a high school education vs. have a college education or greater; Supplementary Table S4).

**TABLE 3:** Stratum-specific associations between nutrients and fatigue for those with (*n* = 272) and without (*n* = 3,819) a history of cancer (except nonmelanoma skin cancer).

	Cancer survivors				Those without a history of cancer			
Parameters	OR	Lower 95% confidence limit	Upper 95% confidence limit	*P*	OR	Lower 95% confidence limit	Upper 95% confidence limit	*P*
α-Carotene	0.988	0.954	1.024	0.48	0.997	0.986	1.008	0.58
*trans*-β-Carotene	1.000	0.990	1.011	0.95	1.000	0.995	1.005	1.00
*cis*-β-Carotene	1.029	0.882	1.200	0.70	0.999	0.901	1.107	0.98
β-Cryptoxanthin	0.991	0.941	1.043	0.70	1.002	0.997	1.008	0.35
γ-Tocopherol	1.001	0.999	1.003	0.20	1.000	1.000	1.001	0.13
Lutein and zeaxanthin	0.970	0.932	1.009	0.12	0.997	0.989	1.006	0.54
*trans*-Lycopene	0.981	0.952	1.010	0.18	0.992	0.984	0.999	0.03^a^
Retinyl palmitate	0.925	0.796	1.075	0.29	0.969	0.930	1.010	0.12
Retinyl stearate	0.792	0.475	1.318	0.34	0.880	0.767	1.011	0.07
Vitamin A	1.004	0.993	1.015	0.47	0.999	0.994	1.005	0.83
Vitamin E	1.000	1.000	1.001	0.52	1.000	1.000	1.000	0.99
Total (*cis*- and *trans*-) lycopene	0.993	0.977	1.009	0.34	0.996	0.991	1.000	0.07

NOTE: Models are adjusted for age, body mass index, race/ethnicity, education, physical activity, history of a cancer diagnosis, and nutrient concentration. Adjusted ORs and 95% CIs are reported.

^a^ *P* < 0.05.

## Discussion

Herein, higher serum concentrations of carotenoids, vitamin A, and vitamin E, which correlate with fruit and vegetable intake, were collectively associated with lower odds of greater fatigue (Fig. 1; Table 2). These data were derived from a nationally representative sample of Americans, with racial and ethnic minorities oversampled and, although these data are correlational and hypothesis-generating by nature, they warrant further investigation of dietary interventions that increase serum carotenoid concentrations to address cancer-related fatigue in survivorship.

Carotenoids are high in many fruits and vegetables and are established as reliable biomarkers of fruit and vegetable intake (21). Thus, our results that higher serum carotenoid concentrations were associated with less fatigue (Table 2) suggest that higher fruit and vegetable intake are associated with less fatigue. These results are consistent with Beydoun and colleagues (26), who observed that lower lutein + zeaxanthin concentrations were associated with poor sleep-related daytime dysfunction in a different subset of the 2005–2006 NHANES cohort (controlling for cancer diagnosis). In addition, a recent meta-analysis concluded that a diet rich in fruits and vegetables may alleviate cancer-related fatigue (40). However, Zick and colleagues (15) saw no associations between change in carotenoids and change in fatigue in a randomized controlled trial assessing the effects of a 3-month dietary intervention on fatigue compared to an attention control among 30 breast cancer survivors; they did observe improvements in fatigue.

We interpret the implications of our observed serum carotenoids using total lycopene as an example. We observed that a 21-μg/dL greater concentration of total lycopene (one SD) was associated with an 8.8% lower risk of more severe fatigue (Fig. 1). With directed approaches, this amount of increase in lycopene is feasible with dietary intervention. For example, Zick and colleagues (15) implemented a custom “Fatigue Reduction Diet” that was rich in fruits and vegetables among fatigued breast cancer survivors who consumed <5.5 servings of fruits and vegetables per day. On average, the 3-month dietary intervention increased lycopene concentrations 38 ng/mL (3.8 μg/dL), which was approximately one SD of their observed lycopene distribution. In addition, in the Women's Healthy Eating and Living Study, breast cancer survivors (within four years of primary treatment) were randomized to a control group or a dietary intervention group that encouraged daily consumption of five servings of vegetables, 16 oz of fresh vegetables juice, three servings of fruit, and other dietary practices. Lycopene concentrations increased more than one-SD in 12 months (mean ± SE in approximately 40 participants = 26.1 ± 2.0 at baseline to 35.9 ± 3.1 μg/dL at 12 months; ref. 41).

We observed a statistically significant interaction between cancer and lutein + zeaxanthin; however, among cancer survivors only, there were no statistically significant relationships between cancer and any of the nutrients (Table 3). While this study may not be adequately powered to reveal interactions between cancer and any nutrients, these data suggest that the mechanisms by which nutritional status and fatigue are related may not be specific to the cancer experience. Nevertheless, the prevalence and severity of fatigue is higher among cancer survivors compared with people who have not had a cancer diagnosis (Table 1), and the development of nutritional interventions are warranted to alleviate fatigue among this high-risk population. Mechanisms underlying other chronic fatigue disorders include chronic inflammation, oxidative stress, mitochondrial dysfunction, circadian rhythm disruption, and others (42, 43). Adverse pathophysiology can be initiated or exacerbated by the cancer experience, and cancer-related fatigue can be derived from the pathology of the cancer itself; the biological effects of surgery, radiation, and/or chemotherapy; and the mental burden of a cancer diagnosis and treatment. In addition, after a cancer diagnosis, fatigue, cognitive impairment, negative affect, and sleep disturbances tend to co-occur—the “psychoneurologic symptom cluster;” these symptoms likely share underlying pathologies in the brain and in the periphery (44). Nutritional interventions can effectively reduce inflammation and oxidative stress (45) and are promising in the prevention and treatment of cancer-related fatigue (40).

Because serum carotenoids are biomarkers for higher fruit and vegetable intake, it is unknown if associations between serum carotenoids and fatigue stem from the carotenoids themselves, other components of carotenoid-rich foods (e.g., dietary fiber), and/or synergistic effects between carotenoids and other components of the food matrix (46). While there have been no studies to our knowledge testing the effects of supplementation of carotenoids from dietary supplements on supportive care outcomes, randomized controlled trials testing the effects of β-carotene supplementation on the development or progression of cancer have been null or suggest that supplementation can increase the risk of poor outcomes, especially in current smokers (47). Moving forward, it will be important to determine who an intervention may benefit (e.g., survivors of which type(s) of cancer, which treatments survivors are undergoing/underwent, baseline demographics or metabolic clinical characteristics), components of an optimized intervention (e.g., length in months, which dietary patterns), and whether carotenoids are useful biomarkers for intervention adherence and/or risk for persistent cancer-related fatigue.

Interestingly, we observed that Hispanic participants, especially Mexican Americans, and Black participants had a lower incidence of cancer than White participants (Table 1), and Mexican Americans reported less fatigue (Supplementary Table S1). Our study was not designed to probe why certain races and ethnicities had higher or lower fatigue; furthermore, this literature base is scant. This observation might be spurious because, while NHANES strives to be nationally representative—surveys are in English or Spanish and most of the staff is bilingual—minorities face other barriers to participating in research (48) and the NHANES population might not exactly represent the U.S. population. If our observation is accurate, we hypothesize that Mexican Americans might have had lower fatigue on average in part because of the “Hispanic paradox,”— selective migration leads to Mexican migrants being healthier than the average Mexican, the average U.S.-born Mexican American, and U.S.-born White American (49). With that said, the relationships between race, ethnicity, socioeconomic status, and health status are very complex. With the very little literature on diet and cancer-related fatigue, especially how race, ethnicity, and migration status factor in, it is important to follow up on these relationships in order to optimize efficacy and adherence of dietary interventions for fatigue among diverse populations.

Whole-food nutritional interventions are in their infancy and remain promising to minimize cancer-related fatigue. Exercise and psychosocial behavioral interventions are unequivocally beneficial in the treatment of cancer-related fatigue and, in fact, more potent than current pharmaceutical interventions (4), though nutritional interventions remain understudied (40). During chemotherapy, patients tend to consume less total energy, fat, and protein (50), which leads to a large prevalence of malnutrition, especially among older patients (51, 52). The American Institute for Cancer Research (53) and the American Cancer Society (54) recommend healthy eating patterns in survivorship that are high in fruits, vegetables, and whole grains to bolster health and prevent recurrence; however, no supplements are yet recommended via clinical guidelines that can address cancer-related fatigue (55). In fact, antioxidant supplements (e.g., carotenoids) are contraindicated during chemotherapy treatment, in part because antioxidants could attenuate the efficacy of chemotherapeutic agents or increase the risk of recurrence (55, 56). In addition, it is believed that nutrients from whole foods have synergistic benefits on health that cannot be harnessed from extracts of bioactive compounds (55).

This study has a unique strength in that we were able to assess patient-reported fatigue among thousands of Americans and investigate associations between serum carotenoid concentration, a proxy for diet quality, and fatigue among people with and without a cancer diagnosis. Using ordinal logistic regression modeling, we were able to investigate effect modification of a history of cancer and nutrients on the experience of fatigue in survivorship.

This study, however, is not without limitations. Herein, fatigue was measured using a single-item question regarding feeling tired and having little energy; it was not measured using a standard, validated uni- or multi-dimensional questionnaire (57). However, single-item questions that capture fatigue are very common and have been validated and deemed useful for screening, diagnosis, and research purposes (32, 58). For example, Wolfe observed that the single question, “How much of a problem has fatigue or tiredness been for you in the past week?” with response options 0–10, performed at least as well as longer fatigue questionnaires in regard to sensitivity to change and correlation with clinical measures (31). Also, this study was cross-sectional and therefore we cannot declare causation. Causation could occur in either direction, where good nutritional status could help alleviate fatigue, or having more energy could help facilitate healthy dietary behaviors and/or healthy metabolism. More research is needed to investigate whether interventions that improve diet quality could help alleviate cancer-related fatigue post-diagnosis and post-primary treatment. Moreover, a history of a cancer diagnosis is associated with a plethora of psychological (e.g., stress), logistical (e.g., interference in a person's job and personal life), and biological factors (e.g., chemotherapy, radiation, hormone therapy) that could all contribute to fatigue, and these factors could not be distinguished herein. However, this broad analysis among a heterogeneous survivor population contributes information regarding relationships between carotenoids and cancer-related fatigue that apply to the whole population. Finally, this was a secondary analysis and is therefore hypothesis-generating rather than hypothesis-testing. These results should be confirmed in future studies where assessments of the correlation between carotenoid concentration and fatigue are the primary outcome.

## Conclusions

Associations were observed between circulating carotenoid concentrations (which reflect carotenoid and therefore fruit and vegetable intake) and fatigue in a large population that is generalizable to the American population, though there was no evidence of an association between serum carotenoids and fatigue that was specific to the cancer experience. The high prevalence and severity of fatigue among survivors, in combination with the observation that higher serum carotenoid concentrations were associated with less fatigue, warrants future randomized controlled trials that aim to increase fruit and vegetable intake to reduce the susceptibility to fatigue among cancer survivors.

## Supplementary Material

Supplementary Tables S1-S4 Suppl. Table 1. Results of crude and adjusted models describing the association between carotenoid concentrations and fatigue: all model output. For body mass index, estimates are compared to normal weight (18.5-<25 kg/m2); 1=<18.5 kg/m2, 3=25-<30 kg/m2, and 4=≥30 kg/m2). For race/ethnicity, estimates are compared to non-Hispanic White, 1=Mexican American, 2=Other Hispanic, 4=non-Hispanic Black American, 5=Other non-Hispanic race, including multi-racial. For education, estimates are compared to having at least a college education (11= less than a high school education, 12= at least high school but less than a four-year college education, 13=at least a four-year college education).
Suppl. Table 2. Results of adjusted model with the nutrient×cancer interaction term to explore the associations between carotenoids and cancer-related fatigue: all model output.
Suppl. Table 3. Adjusted odds ratios and 95% confidence intervals for the effects of the nutrient×cancer interaction on fatigue. Models are adjusted for age, body mass index, race/ethnicity, education, physical activity, history of a cancer diagnosis, and nutrient concentration.
Suppl. Table 4. Results of adjusted models (no interaction term) describing the association between carotenoid concentrations and fatigue only among cancer survivors: all model output; estimates for race/ethnicity are compared to non-Hispanic Whites, 1=Mexican American, 2=Other Hispanic, 4=non-Hispanic Black American, 5=Other non-Hispanic race, including multi-racial; estimates for body mass index are compared to those of normal weight; and estimates for education are compared to those with at least a college education. Exercise is estimated from metabolic equivalents (MET hours) per week as a continuous variable. Age, Years since Diagnosis, and carotenoid concentration are treated as continuous variables. (n=272)
