# Supplementary material for: Serum Carotenoids and Cancer-Related Fatigue: An Analysis of the 2005–2006 National Health and Nutrition Examination Survey
Source: Cancer Res Commun. 2022 Mar 31;2(3):202–10. doi: 10.1158/2767-9764.CRC-21-0172 (PMC9489051; doi:10.1158/2767-9764.CRC-21-0172)
Supplement: Supplementary Tables S1-S4 — Suppl. Table 1. Results of crude and adjusted models describing the association between carotenoid concentrations and fatigue: all model output. For body mass index, estimates are compared to normal weight (18.5-<25 kg/m2); 1=<18.5 kg/m2, 3=25-<30 kg/m2, and 4=≥30 kg/m2). For race/ethnicity, estimates are compared to non-Hispanic White, 1=Mexican American, 2=Other Hispanic, 4=non-Hispanic Black American, 5=Other non-Hispanic race, including multi-racial. For education, estimates are compared to having at least a college education (11= less than a high school education, 12= at least high school but less than a four-year college education, 13=at least a four-year college education). Suppl. Table 2. Results of adjusted model with the nutrient×cancer interaction term to explore the associations between carotenoids and cancer-related fatigue: all model output. Suppl. Table 3. Adjusted odds ratios and 95% confidence intervals for the effects of the nutrient×cancer interaction on fatigue. Models are adjusted for age, body mass index, race/ethnicity, education, physical activity, history of a cancer diagnosis, and nutrient concentration. Suppl. Table 4. Results of adjusted models (no interaction term) describing the association between carotenoid concentrations and fatigue only among cancer survivors: all model output; estimates for race/ethnicity are compared to non-Hispanic Whites, 1=Mexican American, 2=Other Hispanic, 4=non-Hispanic Black American, 5=Other non-Hispanic race, including multi-racial; estimates for body mass index are compared to those of normal weight; and estimates for education are compared to those with at least a college education. Exercise is estimated from metabolic equivalents (MET hours) per week as a continuous variable. Age, Years since Diagnosis, and carotenoid concentration are treated as continuous variables. (n=272) [file crc-21-0172-s01.docx]

# Kleckner et al., Supplemental material

## **Suppl. Table 1.** Results of crude and adjusted models describing the association between carotenoid concentrations and fatigue: all model output. For body mass index, estimates are compared to normal weight (18.5-<25 kg/m^2^); 1=<18.5 kg/m^2^, 3=25-<30 kg/m^2^, and 4=≥30 kg/m^2^). For race/ethnicity, estimates are compared to non-Hispanic White, 1=Mexican American, 2=Other Hispanic, 4=non-Hispanic Black American, 5=Other non-Hispanic race, including multi-racial. For education, estimates are compared to having at least a college education (11= less than a high school education, 12= at least high school but less than a four-year college education, 13=at least a four-year college education).

Crude models

| Parameter | | Estimate | Standard | t Value | p-value | 95% Confidence Limits | | Odds ratio estimate | |  |
| --- | --- | --- | --- | --- | --- | --- | --- | --- | --- | --- |
|  |  |  | Error |  |  |  |  | Point Estimate | 95% Confidence Limits | |
| Intercept | 3 | -2.6237 | 0.0823 | -31.88 | <0.0001 | -2.7991 | -2.4483 |  |  |  |
| Intercept | 2 | -1.7788 | 0.0742 | -23.97 | <0.0001 | -1.9369 | -1.6206 |  |  |  |
| Intercept | 1 | 0.0384 | 0.0484 | 0.79 | 0.4396 | -0.0648 | 0.1417 |  |  |  |
| α-Carotene |  | -0.0102 | 0.00453 | -2.26 | 0.0391 | -0.0199 | -0.00058 | 0.989852 | 0.980297 | 0.99942 |
|  |  |  |  |  |  |  |  |  |  |  |
| Parameter | | Estimate | Standard | t Value | p-value | 95% Confidence Limits | | Point Estimate | 95% Confidence Limits | |
|  |  |  | Error |  |  |  |  |  |  |  |
| Intercept | 3 | -2.6287 | 0.0754 | -34.88 | <0.0001 | -2.7894 | -2.4681 |  |  |  |
| Intercept | 2 | -1.7843 | 0.0735 | -24.27 | <0.0001 | -1.941 | -1.6277 |  |  |  |
| Intercept | 1 | 0.034 | 0.0554 | 0.61 | 0.5485 | -0.0841 | 0.1522 |  |  |  |
| trans-β-Carotene |  | -0.00235 | 0.00191 | -1.23 | 0.2376 | -0.00642 | 0.00172 | 0.997653 | 0.993601 | 1.001721 |
|  |  |  |  |  |  |  |  |  |  |  |
| Parameter | | Estimate | Standard | t Value | p-value | 95% Confidence Limits | | Point Estimate | 95% Confidence Limits | |
|  |  |  | Error |  |  |  |  |  |  |  |
| Intercept | 3 | -2.6272 | 0.0755 | -34.8 | <0.0001 | -2.7881 | -2.4663 |  |  |  |
| Intercept | 2 | -1.7665 | 0.0823 | -21.46 | <0.0001 | -1.9419 | -1.591 |  |  |  |
| Intercept | 1 | 0.0532 | 0.0612 | 0.87 | 0.3983 | -0.0772 | 0.1836 |  |  |  |
| cis-β-Carotene |  | -0.0395 | 0.0339 | -1.16 | 0.2627 | -0.1117 | 0.0328 | 0.96127 | 0.894313 | 1.033344 |
|  |  |  |  |  |  |  |  |  |  |  |
| Parameter | | Estimate | Standard | t Value | p-value | 95% Confidence Limits | | Point Estimate | 95% Confidence Limits | |
|  |  |  | Error |  |  |  |  |  |  |  |
| Intercept | 3 | -2.6314 | 0.0866 | -30.37 | <0.0001 | -2.8161 | -2.4467 |  |  |  |
| Intercept | 2 | -1.7854 | 0.0773 | -23.09 | <0.0001 | -1.9502 | -1.6206 |  |  |  |
| Intercept | 1 | 0.0314 | 0.047 | 0.67 | 0.5143 | -0.0687 | 0.1315 |  |  |  |
| β-Cryptoxanthin |  | -0.00382 | 0.00358 | -1.07 | 0.3028 | -0.0115 | 0.00381 | 0.996187 | 0.988566 | 1.003817 |
|  |  |  |  |  |  |  |  |  |  |  |
| Parameter | | Estimate | Standard | t Value | p-value | 95% Confidence Limits | | Point Estimate | 95% Confidence Limits | |
|  |  |  | Error |  |  |  |  |  |  |  |
| Intercept | 3 | -2.8705 | 0.0997 | -28.78 | <0.0001 | -3.083 | -2.6579 |  |  |  |
| Intercept | 2 | -2.0216 | 0.1014 | -19.94 | <0.0001 | -2.2377 | -1.8054 |  |  |  |
| Intercept | 1 | -0.1984 | 0.0735 | -2.7 | 0.0165 | -0.355 | -0.0417 |  |  |  |
| γ-Tocopherol |  | 0.000843 | 0.000301 | 2.81 | 0.0133 | 0.000203 | 0.00148 | 1.000843 | 1.000203 | 1.001481 |
|  |  |  |  |  |  |  |  |  |  |  |
| Parameter | | Estimate | Standard | t Value | p-value | 95% Confidence Limits | | Point Estimate | 95% Confidence Limits | |
|  |  |  | Error |  |  |  |  |  |  |  |
| Intercept | 3 | -2.5281 | 0.1018 | -24.82 | <0.0001 | -2.7452 | -2.311 |  |  |  |
| Intercept | 2 | -1.6829 | 0.0866 | -19.44 | <0.0001 | -1.8674 | -1.4983 |  |  |  |
| Intercept | 1 | 0.1367 | 0.0544 | 2.51 | 0.024 | 0.0206 | 0.2527 |  |  |  |
| Lutein and zeaxanthin |  | -0.00892 | 0.00314 | -2.84 | 0.0124 | -0.0156 | -0.00223 | 0.99112 | 0.984521 | 0.997772 |
|  |  |  |  |  |  |  |  |  |  |  |
| Parameter | | Estimate | Standard | t Value | p-value | 95% Confidence Limits | | Point Estimate | 95% Confidence Limits | |
|  |  |  | Error |  |  |  |  |  |  |  |
| Intercept | 3 | -2.4608 | 0.0798 | -30.84 | <0.0001 | -2.6309 | -2.2908 |  |  |  |
| Intercept | 2 | -1.6151 | 0.0964 | -16.75 | <0.0001 | -1.8207 | -1.4095 |  |  |  |
| Intercept | 1 | 0.2053 | 0.081 | 2.53 | 0.0229 | 0.0326 | 0.3781 |  |  |  |
| trans-Lycopene |  | -0.00888 | 0.00321 | -2.76 | 0.0145 | -0.0157 | -0.00203 | 0.991159 | 0.984423 | 0.997972 |
|  |  |  |  |  |  |  |  |  |  |  |
| Parameter | | Estimate | Standard | t Value | p-value | 95% Confidence Limits | | Point Estimate | 95% Confidence Limits | |
|  |  |  | Error |  |  |  |  |  |  |  |
| Intercept | 3 | -2.5528 | 0.0778 | -32.82 | <0.0001 | -2.7186 | -2.3871 |  |  |  |
| Intercept | 2 | -1.6987 | 0.0664 | -25.58 | <0.0001 | -1.8402 | -1.5571 |  |  |  |
| Intercept | 1 | 0.1318 | 0.056 | 2.35 | 0.0327 | 0.0124 | 0.2512 |  |  |  |
| Retinyl palmitate |  | -0.0532 | 0.0148 | -3.6 | 0.0026 | -0.0846 | -0.0217 | 0.94819 | 0.91888 | 0.978534 |
|  |  |  |  |  |  |  |  |  |  |  |
| Parameter | | Estimate | Standard | t Value | p-value | 95% Confidence Limits | | Point Estimate | 95% Confidence Limits | |
|  |  |  | Error |  |  |  |  |  |  |  |
| Intercept | 3 | -2.5193 | 0.0796 | -31.67 | <0.0001 | -2.6889 | -2.3498 |  |  |  |
| Intercept | 2 | -1.6915 | 0.0671 | -25.23 | <0.0001 | -1.8345 | -1.5486 |  |  |  |
| Intercept | 1 | 0.127 | 0.0578 | 2.2 | 0.0443 | 0.00371 | 0.2502 |  |  |  |
| Retinyl stearate |  | -0.1943 | 0.0574 | -3.38 | 0.0041 | -0.3167 | -0.0719 | 0.823411 | 0.728549 | 0.930624 |
|  |  |  |  |  |  |  |  |  |  |  |
| Parameter | | Estimate | Standard | t Value | p-value | 95% Confidence Limits | | Point Estimate | 95% Confidence Limits | |
|  |  |  | Error |  |  |  |  |  |  |  |
| Intercept | 3 | -2.5308 | 0.1297 | -19.51 | <0.0001 | -2.8073 | -2.2543 |  |  |  |
| Intercept | 2 | -1.6868 | 0.1235 | -13.66 | <0.0001 | -1.9501 | -1.4235 |  |  |  |
| Intercept | 1 | 0.1313 | 0.1406 | 0.93 | 0.3649 | -0.1682 | 0.4309 |  |  |  |
| Vitamin A |  | -0.00236 | 0.0022 | -1.07 | 0.2999 | -0.00704 | 0.00232 | 0.997643 | 0.992985 | 1.002323 |
|  |  |  |  |  |  |  |  |  |  |  |
| Parameter | | Estimate | Standard | t Value | p-value | 95% Confidence Limits | | Point Estimate | 95% Confidence Limits | |
|  |  |  | Error |  |  |  |  |  |  |  |
| Intercept | 3 | -2.5897 | 0.0941 | -27.53 | <0.0001 | -2.7902 | -2.3892 |  |  |  |
| Intercept | 2 | -1.7455 | 0.0788 | -22.16 | <0.0001 | -1.9134 | -1.5776 |  |  |  |
| Intercept | 1 | 0.0724 | 0.0763 | 0.95 | 0.3574 | -0.0901 | 0.235 |  |  |  |
| Vitamin E |  | -0.00007 | 0.000062 | -1.07 | 0.3 | -0.0002 | 0.000065 | 0.99993 | 0.9998 | 1.000065 |
|  |  |  |  |  |  |  |  |  |  |  |
| Parameter | | Estimate | Standard | t Value | p-value | 95% Confidence Limits | | Point Estimate | 95% Confidence Limits | |
|  |  |  | Error |  |  |  |  |  |  |  |
| Intercept | 3 | -2.4821 | 0.0755 | -32.87 | <0.0001 | -2.643 | -2.3211 |  |  |  |
| Intercept | 2 | -1.6273 | 0.1003 | -16.23 | <0.0001 | -1.841 | -1.4136 |  |  |  |
| Intercept | 1 | 0.1941 | 0.0871 | 2.23 | 0.0417 | 0.00836 | 0.3797 |  |  |  |
| Total lycopene |  | -0.0044 | 0.00188 | -2.34 | 0.0334 | -0.00841 | -0.0004 | 0.99561 | 0.991625 | 0.9996 |

Adjusted models

| Parameter | | Estimate | Standard | t Value | p-value | 95% Confidence Limits | | Odds ratio estimates | |  |
| --- | --- | --- | --- | --- | --- | --- | --- | --- | --- | --- |
|  |  |  | Error |  |  |  |  | Point Estimate | 95% Confidence Limits | |
| Intercept | 3 | -2.3315 | 0.1421 | -16.41 | <0.0001 | -2.6343 | -2.0287 |  |  |  |
| Intercept | 2 | -1.4868 | 0.1152 | -12.91 | <0.0001 | -1.7322 | -1.2413 |  |  |  |
| Intercept | 1 | 0.3621 | 0.1257 | 2.88 | 0.0114 | 0.0942 | 0.6299 |  |  |  |
| Age |  | -0.00761 | 0.00229 | -3.32 | 0.0047 | -0.0125 | -0.00272 | 0.992 | 0.988 | 0.997 |
| Race/ethnicity | 1 | -0.3208 | 0.1212 | -2.65 | 0.0183 | -0.5791 | -0.0624 | 0.726 | 0.56 | 0.94 |
| Race/ethnicity | 2 | 0.0355 | 0.264 | 0.13 | 0.8948 | -0.5272 | 0.5982 | 1.036 | 0.59 | 1.819 |
| Race/ethnicity | 4 | -0.0565 | 0.1188 | -0.48 | 0.6412 | -0.3098 | 0.1967 | 0.945 | 0.734 | 1.217 |
| Race/ethnicity | 5 | 0.3035 | 0.207 | 1.47 | 0.1632 | -0.1376 | 0.7447 | 1.355 | 0.871 | 2.106 |
| Education | . | 1.0014 | 0.1006 | 9.95 | <0.0001 | 0.7869 | 1.2158 | 2.722 | 2.197 | 3.373 |
| Education | 11 | 0.1413 | 0.1149 | 1.23 | 0.2378 | -0.1037 | 0.3862 | 1.152 | 0.902 | 1.471 |
| Education | 12 | -0.00227 | 0.097 | -0.02 | 0.9817 | -0.209 | 0.2045 | 0.998 | 0.811 | 1.227 |
| Body mass index | 1 | 0.1277 | 0.2546 | 0.5 | 0.6232 | -0.415 | 0.6704 | 1.136 | 0.66 | 1.955 |
| Body mass index | 3 | -0.0436 | 0.0873 | -0.5 | 0.6246 | -0.2297 | 0.1425 | 0.957 | 0.795 | 1.153 |
| Body mass index | 4 | 0.2444 | 0.1169 | 2.09 | 0.054 | -0.00479 | 0.4936 | 1.277 | 0.995 | 1.638 |
| Exercise |  | -0.00593 | 0.0012 | -4.94 | 0.0002 | -0.00849 | -0.00337 | 0.994 | 0.992 | 0.997 |
| Cancer (y/n) | y | 0.3649 | 0.1553 | 2.35 | 0.0329 | 0.034 | 0.6958 | 1.44 | 1.035 | 2.005 |
| α-Carotene |  | -0.00396 | 0.00458 | -0.86 | 0.4009 | -0.0137 | 0.0058 | 0.996048 | 0.986393 | 1.005817 |
|  |  |  |  |  |  |  |  |  |  |  |
| Parameter | | Estimate | Standard | t Value | p-value | 95% Confidence Limits | | Point Estimate | 95% Confidence Limits | |
|  |  |  | Error |  |  |  |  |  |  |  |
| Intercept | 3 | -2.3513 | 0.1429 | -16.46 | <0.0001 | -2.6558 | -2.0468 |  |  |  |
| Intercept | 2 | -1.5069 | 0.1199 | -12.56 | <0.0001 | -1.7626 | -1.2513 |  |  |  |
| Intercept | 1 | 0.3433 | 0.132 | 2.6 | 0.0201 | 0.0619 | 0.6248 |  |  |  |
| Age |  | -0.00783 | 0.00221 | -3.54 | 0.003 | -0.0125 | -0.00311 | 0.992 | 0.988 | 0.997 |
| Race/ethnicity | 1 | -0.3259 | 0.1221 | -2.67 | 0.0175 | -0.5861 | -0.0657 | 0.722 | 0.556 | 0.936 |
| Race/ethnicity | 2 | 0.0228 | 0.2595 | 0.09 | 0.9311 | -0.5304 | 0.576 | 1.023 | 0.588 | 1.779 |
| Race/ethnicity | 4 | -0.0556 | 0.1189 | -0.47 | 0.6465 | -0.3091 | 0.1978 | 0.946 | 0.734 | 1.219 |
| Race/ethnicity | 5 | 0.302 | 0.2115 | 1.43 | 0.1738 | -0.1488 | 0.7528 | 1.353 | 0.862 | 2.123 |
| Education | . | 1.0207 | 0.0999 | 10.21 | <0.0001 | 0.8077 | 1.2337 | 2.775 | 2.243 | 3.434 |
| Education | 11 | 0.154 | 0.1235 | 1.25 | 0.2318 | -0.1094 | 0.4173 | 1.166 | 0.896 | 1.518 |
| Education | 12 | 0.00644 | 0.1024 | 0.06 | 0.9507 | -0.2118 | 0.2247 | 1.006 | 0.809 | 1.252 |
| Body mass index | 1 | 0.1288 | 0.2542 | 0.51 | 0.6198 | -0.4131 | 0.6707 | 1.137 | 0.662 | 1.956 |
| Body mass index | 3 | -0.0372 | 0.0884 | -0.42 | 0.6799 | -0.2257 | 0.1513 | 0.963 | 0.798 | 1.163 |
| Body mass index | 4 | 0.2548 | 0.1139 | 2.24 | 0.0409 | 0.012 | 0.4977 | 1.29 | 1.012 | 1.645 |
| Exercise |  | -0.00597 | 0.00122 | -4.91 | 0.0002 | -0.00857 | -0.00338 | 0.994 | 0.991 | 0.997 |
| Cancer (y/n) | y | 0.3654 | 0.1563 | 2.34 | 0.0337 | 0.0322 | 0.6986 | 1.441 | 1.033 | 2.011 |
| trans-β-Carotene |  | -0.00008 | 0.00212 | -0.04 | 0.9711 | -0.0046 | 0.00444 | 0.99992 | 0.995411 | 1.00445 |
|  |  |  |  |  |  |  |  |  |  |  |
| Parameter | | Estimate | Standard | t Value | p-value | 95% Confidence Limits | | Point Estimate | 95% Confidence Limits | |
|  |  |  | Error |  |  |  |  |  |  |  |
| Intercept | 3 | -2.3999 | 0.1417 | -16.94 | <0.0001 | -2.7019 | -2.0979 |  |  |  |
| Intercept | 2 | -1.5386 | 0.1391 | -11.06 | <0.0001 | -1.8352 | -1.242 |  |  |  |
| Intercept | 1 | 0.3153 | 0.158 | 1.99 | 0.0646 | -0.0216 | 0.6521 |  |  |  |
| Age |  | -0.00783 | 0.00233 | -3.36 | 0.0043 | -0.0128 | -0.00287 | 0.992 | 0.987 | 0.997 |
| Race/ethnicity | 1 | -0.3243 | 0.141 | -2.3 | 0.0362 | -0.6248 | -0.0238 | 0.723 | 0.535 | 0.977 |
| Race/ethnicity | 2 | -0.00919 | 0.2759 | -0.03 | 0.9739 | -0.5973 | 0.5789 | 0.991 | 0.55 | 1.784 |
| Race/ethnicity | 4 | -0.0997 | 0.1218 | -0.82 | 0.4261 | -0.3593 | 0.16 | 0.905 | 0.698 | 1.174 |
| Race/ethnicity | 5 | 0.2914 | 0.1822 | 1.6 | 0.1307 | -0.0971 | 0.6798 | 1.338 | 0.908 | 1.974 |
| Education | . | 1.0417 | 0.119 | 8.75 | <0.0001 | 0.788 | 1.2954 | 2.834 | 2.199 | 3.653 |
| Education | 11 | 0.1802 | 0.1243 | 1.45 | 0.1676 | -0.0847 | 0.4451 | 1.197 | 0.919 | 1.561 |
| Education | 12 | 0.0647 | 0.1102 | 0.59 | 0.5659 | -0.1702 | 0.2995 | 1.067 | 0.844 | 1.349 |
| Body mass index | 1 | 0.0772 | 0.2573 | 0.3 | 0.7682 | -0.4713 | 0.6257 | 1.08 | 0.624 | 1.87 |
| Body mass index | 3 | -0.0182 | 0.0917 | -0.2 | 0.8457 | -0.2137 | 0.1774 | 0.982 | 0.808 | 1.194 |
| Body mass index | 4 | 0.274 | 0.1228 | 2.23 | 0.0414 | 0.0121 | 0.5358 | 1.315 | 1.012 | 1.709 |
| Exercise |  | -0.00614 | 0.00129 | -4.76 | 0.0003 | -0.00889 | -0.00339 | 0.994 | 0.991 | 0.997 |
| Cancer (y/n) | y | 0.3943 | 0.1601 | 2.46 | 0.0264 | 0.0531 | 0.7355 | 1.483 | 1.055 | 2.087 |
| cis-β-Carotene |  | 0.00112 | 0.0396 | 0.03 | 0.9777 | -0.0833 | 0.0855 | 1.001121 | 0.920075 | 1.089262 |
|  |  |  |  |  |  |  |  |  |  |  |
| Parameter | | Estimate | Standard | t Value | p-value | 95% Confidence Limits | | Point Estimate | 95% Confidence Limits | |
|  |  |  | Error |  |  |  |  |  |  |  |
| Intercept | 3 | -2.3758 | 0.1467 | -16.2 | <0.0001 | -2.6885 | -2.0632 |  |  |  |
| Intercept | 2 | -1.5295 | 0.1195 | -12.8 | <0.0001 | -1.7841 | -1.2749 |  |  |  |
| Intercept | 1 | 0.3201 | 0.1272 | 2.52 | 0.0237 | 0.0491 | 0.5912 |  |  |  |
| Age |  | -0.00792 | 0.00234 | -3.38 | 0.0041 | -0.0129 | -0.00293 | 0.992 | 0.987 | 0.997 |
| Race/ethnicity | 1 | -0.3578 | 0.1232 | -2.9 | 0.0109 | -0.6204 | -0.0952 | 0.699 | 0.538 | 0.909 |
| Race/ethnicity | 2 | 0.00818 | 0.255 | 0.03 | 0.9748 | -0.5353 | 0.5516 | 1.008 | 0.586 | 1.736 |
| Race/ethnicity | 4 | -0.0624 | 0.1185 | -0.53 | 0.6063 | -0.3149 | 0.1902 | 0.94 | 0.73 | 1.209 |
| Race/ethnicity | 5 | 0.2839 | 0.2026 | 1.4 | 0.1814 | -0.1479 | 0.7157 | 1.328 | 0.863 | 2.046 |
| Body mass index | 1 | 0.1248 | 0.2527 | 0.49 | 0.6284 | -0.4137 | 0.6634 | 1.133 | 0.661 | 1.941 |
| Body mass index | 3 | -0.0263 | 0.0858 | -0.31 | 0.7636 | -0.2093 | 0.1567 | 0.974 | 0.811 | 1.17 |
| Body mass index | 4 | 0.2618 | 0.1184 | 2.21 | 0.043 | 0.00936 | 0.5143 | 1.299 | 1.009 | 1.672 |
| Education | . | 1.0277 | 0.1164 | 8.83 | <0.0001 | 0.7796 | 1.2758 | 2.795 | 2.181 | 3.581 |
| Education | 11 | 0.1623 | 0.1093 | 1.49 | 0.158 | -0.0705 | 0.3952 | 1.176 | 0.932 | 1.485 |
| Education | 12 | 0.023 | 0.0894 | 0.26 | 0.8008 | -0.1676 | 0.2135 | 1.023 | 0.846 | 1.238 |
| Exercise |  | -0.00609 | 0.00117 | -5.22 | 0.0001 | -0.00858 | -0.00361 | 0.994 | 0.991 | 0.996 |
| Cancer (y/n) | y | 0.3752 | 0.1559 | 2.41 | 0.0294 | 0.0429 | 0.7076 | 1.455 | 1.044 | 2.029 |
| β-Cryptoxanthin |  | 0.00215 | 0.00285 | 0.75 | 0.4627 | -0.00393 | 0.00823 | 1.002152 | 0.996078 | 1.008264 |
|  |  |  |  |  |  |  |  |  |  |  |
| Parameter | | Estimate | Standard | t Value | p-value | 95% Confidence Limits | | Point Estimate | 95% Confidence Limits | |
|  |  |  | Error |  |  |  |  |  |  |  |
| Intercept | 3 | -2.4606 | 0.1164 | -21.15 | <0.0001 | -2.7086 | -2.2126 |  |  |  |
| Intercept | 2 | -1.6121 | 0.0984 | -16.38 | <0.0001 | -1.8219 | -1.4023 |  |  |  |
| Intercept | 1 | 0.2405 | 0.1017 | 2.36 | 0.032 | 0.0236 | 0.4573 |  |  |  |
| Age |  | -0.00741 | 0.00227 | -3.27 | 0.0052 | -0.0122 | -0.00258 | 0.993 | 0.988 | 0.997 |
| Race/ethnicity | 1 | -0.3219 | 0.1213 | -2.65 | 0.0181 | -0.5805 | -0.0633 | 0.725 | 0.56 | 0.939 |
| Race/ethnicity | 2 | 0.0332 | 0.2583 | 0.13 | 0.8994 | -0.5173 | 0.5838 | 1.034 | 0.596 | 1.793 |
| Race/ethnicity | 4 | -0.0506 | 0.1186 | -0.43 | 0.6759 | -0.3034 | 0.2022 | 0.951 | 0.738 | 1.224 |
| Race/ethnicity | 5 | 0.2958 | 0.2025 | 1.46 | 0.1647 | -0.1358 | 0.7275 | 1.344 | 0.873 | 2.07 |
| Body mass index | 1 | 0.1258 | 0.2519 | 0.5 | 0.6248 | -0.4112 | 0.6628 | 1.134 | 0.663 | 1.94 |
| Body mass index | 3 | -0.0634 | 0.0842 | -0.75 | 0.463 | -0.2429 | 0.1161 | 0.939 | 0.784 | 1.123 |
| Body mass index | 4 | 0.2015 | 0.1162 | 1.73 | 0.1034 | -0.0462 | 0.4492 | 1.223 | 0.955 | 1.567 |
| Education | . | 1.0171 | 0.1017 | 10 | <0.0001 | 0.8004 | 1.2338 | 2.765 | 2.226 | 3.434 |
| Education | 11 | 0.1385 | 0.1159 | 1.2 | 0.2506 | -0.1085 | 0.3855 | 1.149 | 0.897 | 1.47 |
| Education | 12 | -0.00942 | 0.0934 | -0.1 | 0.9211 | -0.2086 | 0.1898 | 0.991 | 0.812 | 1.209 |
| Exercise |  | -0.00578 | 0.00119 | -4.85 | 0.0002 | -0.00832 | -0.00324 | 0.994 | 0.992 | 0.997 |
| Cancer (y/n) | y | 0.374 | 0.16 | 2.34 | 0.0337 | 0.033 | 0.7151 | 1.454 | 1.034 | 2.044 |
| γ-Tocopherol |  | 0.00052 | 0.000273 | 1.91 | 0.0758 | -0.00006 | 0.0011 | 1.00052 | 0.99994 | 1.001101 |
|  |  |  |  |  |  |  |  |  |  |  |
| Parameter | | Estimate | Standard | t Value | p-value | 95% Confidence Limits | | Point Estimate | 95% Confidence Limits | |
|  |  |  | Error |  |  |  |  |  |  |  |
| Intercept | 3 | -2.2871 | 0.1679 | -13.62 | <0.0001 | -2.645 | -1.9292 |  |  |  |
| Intercept | 2 | -1.4422 | 0.1388 | -10.39 | <0.0001 | -1.738 | -1.1463 |  |  |  |
| Intercept | 1 | 0.4085 | 0.1442 | 2.83 | 0.0126 | 0.1012 | 0.7158 |  |  |  |
| Age |  | -0.00735 | 0.00239 | -3.07 | 0.0077 | -0.0124 | -0.00226 | 0.993 | 0.988 | 0.998 |
| Race/ethnicity | 1 | -0.3077 | 0.1249 | -2.46 | 0.0263 | -0.5738 | -0.0416 | 0.735 | 0.563 | 0.959 |
| Race/ethnicity | 2 | 0.0413 | 0.2602 | 0.16 | 0.876 | -0.5132 | 0.5958 | 1.042 | 0.599 | 1.815 |
| Race/ethnicity | 4 | -0.0416 | 0.1179 | -0.35 | 0.7293 | -0.2928 | 0.2097 | 0.959 | 0.746 | 1.233 |
| Race/ethnicity | 5 | 0.3189 | 0.2071 | 1.54 | 0.1444 | -0.1226 | 0.7604 | 1.376 | 0.885 | 2.139 |
| Body mass index | 1 | 0.1406 | 0.2459 | 0.57 | 0.5761 | -0.3836 | 0.6648 | 1.151 | 0.681 | 1.944 |
| Body mass index | 3 | -0.0421 | 0.0896 | -0.47 | 0.6455 | -0.2331 | 0.1489 | 0.959 | 0.792 | 1.161 |
| Body mass index | 4 | 0.2408 | 0.1223 | 1.97 | 0.0677 | -0.0199 | 0.5015 | 1.272 | 0.98 | 1.651 |
| Education | . | 0.9917 | 0.092 | 10.78 | <0.0001 | 0.7956 | 1.1877 | 2.696 | 2.216 | 3.28 |
| Education | 11 | 0.1366 | 0.1166 | 1.17 | 0.2594 | -0.1118 | 0.3851 | 1.146 | 0.894 | 1.47 |
| Education | 12 | -0.0119 | 0.099 | -0.12 | 0.9063 | -0.2229 | 0.1992 | 0.988 | 0.8 | 1.22 |
| Exercise |  | -0.00589 | 0.00122 | -4.83 | 0.0002 | -0.0085 | -0.00329 | 0.994 | 0.992 | 0.997 |
| Cancer (y/n) | y | 0.3628 | 0.155 | 2.34 | 0.0335 | 0.0324 | 0.6932 | 1.437 | 1.033 | 2 |
| Lutein and zeaxanthin |  | -0.00451 | 0.00383 | -1.18 | 0.2574 | -0.0127 | 0.00366 | 0.9955 | 0.98738 | 1.003667 |
|  |  |  |  |  |  |  |  |  |  |  |
| Parameter | | Estimate | Standard | t Value | p-value | 95% Confidence Limits | | Point Estimate | 95% Confidence Limits | |
|  |  |  | Error |  |  |  |  |  |  |  |
| Intercept | 3 | -2.0633 | 0.1915 | -10.77 | <0.0001 | -2.4715 | -1.6551 |  |  |  |
| Intercept | 2 | -1.2174 | 0.1871 | -6.51 | <0.0001 | -1.6162 | -0.8185 |  |  |  |
| Intercept | 1 | 0.6361 | 0.1983 | 3.21 | 0.0059 | 0.2134 | 1.0588 |  |  |  |
| Age |  | -0.00917 | 0.00263 | -3.49 | 0.0033 | -0.0148 | -0.00357 | 0.991 | 0.985 | 0.996 |
| Race/ethnicity | 1 | -0.3551 | 0.1179 | -3.01 | 0.0088 | -0.6064 | -0.1038 | 0.701 | 0.545 | 0.901 |
| Race/ethnicity | 2 | 0.0205 | 0.2599 | 0.08 | 0.9383 | -0.5335 | 0.5745 | 1.021 | 0.587 | 1.776 |
| Race/ethnicity | 4 | -0.053 | 0.1193 | -0.44 | 0.663 | -0.3072 | 0.2012 | 0.948 | 0.735 | 1.223 |
| Race/ethnicity | 5 | 0.2883 | 0.2032 | 1.42 | 0.1764 | -0.1448 | 0.7214 | 1.334 | 0.865 | 2.057 |
| Body mass index | 1 | 0.098 | 0.2657 | 0.37 | 0.7174 | -0.4684 | 0.6644 | 1.103 | 0.626 | 1.943 |
| Body mass index | 3 | -0.0252 | 0.088 | -0.29 | 0.7789 | -0.2127 | 0.1624 | 0.975 | 0.808 | 1.176 |
| Body mass index | 4 | 0.2551 | 0.1173 | 2.18 | 0.046 | 0.00513 | 0.5051 | 1.291 | 1.005 | 1.657 |
| Education | . | 0.8892 | 0.1525 | 5.83 | <0.0001 | 0.5641 | 1.2142 | 2.433 | 1.758 | 3.368 |
| Education | 11 | 0.1217 | 0.1158 | 1.05 | 0.3099 | -0.1252 | 0.3686 | 1.129 | 0.882 | 1.446 |
| Education | 12 | -0.00202 | 0.0968 | -0.02 | 0.9836 | -0.2083 | 0.2042 | 0.998 | 0.812 | 1.227 |
| Exercise |  | -0.00591 | 0.00115 | -5.16 | 0.0001 | -0.00836 | -0.00347 | 0.994 | 0.992 | 0.997 |
| Cancer (y/n) | y | 0.3556 | 0.1528 | 2.33 | 0.0344 | 0.0299 | 0.6814 | 1.427 | 1.03 | 1.977 |
| trans-Lycopene |  | -0.00913 | 0.00389 | -2.35 | 0.033 | -0.0174 | -0.00084 | 0.990912 | 0.982751 | 0.99916 |
|  |  |  |  |  |  |  |  |  |  |  |
| Parameter | | Estimate | Standard | t Value | p-value | 95% Confidence Limits | | Point Estimate | 95% Confidence Limits | |
|  |  |  | Error |  |  |  |  |  |  |  |
| Intercept | 3 | -2.2996 | 0.1733 | -13.27 | <0.0001 | -2.669 | -1.9302 |  |  |  |
| Intercept | 2 | -1.4465 | 0.1431 | -10.11 | <0.0001 | -1.7516 | -1.1415 |  |  |  |
| Intercept | 1 | 0.4141 | 0.1547 | 2.68 | 0.0172 | 0.0843 | 0.7438 |  |  |  |
| Age |  | -0.00714 | 0.0025 | -2.85 | 0.0121 | -0.0125 | -0.0018 | 0.993 | 0.988 | 0.998 |
| Race/ethnicity | 1 | -0.3031 | 0.1087 | -2.79 | 0.0138 | -0.5348 | -0.0714 | 0.739 | 0.586 | 0.931 |
| Race/ethnicity | 2 | 0.0168 | 0.2502 | 0.07 | 0.9473 | -0.5164 | 0.55 | 1.017 | 0.597 | 1.733 |
| Race/ethnicity | 4 | -0.0571 | 0.1156 | -0.49 | 0.6283 | -0.3036 | 0.1893 | 0.944 | 0.738 | 1.208 |
| Race/ethnicity | 5 | 0.2796 | 0.2082 | 1.34 | 0.1993 | -0.1642 | 0.7235 | 1.323 | 0.849 | 2.062 |
| Body mass index | 1 | 0.1025 | 0.2628 | 0.39 | 0.7021 | -0.4576 | 0.6625 | 1.108 | 0.633 | 1.94 |
| Body mass index | 3 | -0.0119 | 0.0897 | -0.13 | 0.8959 | -0.2031 | 0.1792 | 0.988 | 0.816 | 1.196 |
| Body mass index | 4 | 0.2667 | 0.1205 | 2.21 | 0.0428 | 0.00982 | 0.5235 | 1.306 | 1.01 | 1.688 |
| Education | . | 0.9353 | 0.1065 | 8.78 | <0.0001 | 0.7084 | 1.1623 | 2.548 | 2.031 | 3.197 |
| Education | 11 | 0.123 | 0.1216 | 1.01 | 0.3277 | -0.1361 | 0.3821 | 1.131 | 0.873 | 1.465 |
| Education | 12 | -0.0157 | 0.1073 | -0.15 | 0.8855 | -0.2445 | 0.213 | 0.984 | 0.783 | 1.237 |
| Exercise |  | -0.00561 | 0.0012 | -4.66 | 0.0003 | -0.00818 | -0.00305 | 0.994 | 0.992 | 0.997 |
| Cancer (y/n) | y | 0.3079 | 0.1842 | 1.67 | 0.1155 | -0.0848 | 0.7006 | 1.361 | 0.919 | 2.015 |
| Retinyl palmitate |  | -0.0372 | 0.0168 | -2.21 | 0.0433 | -0.073 | -0.00128 | 0.963483 | 0.929601 | 0.998721 |
|  |  |  |  |  |  |  |  |  |  |  |
| Parameter | | Estimate | Standard | t Value | p-value | 95% Confidence Limits | | Point Estimate | 95% Confidence Limits | |
|  |  |  | Error |  |  |  |  |  |  |  |
| Intercept | 3 | -2.2419 | 0.1308 | -17.13 | <0.0001 | -2.5207 | -1.963 |  |  |  |
| Intercept | 2 | -1.4187 | 0.1058 | -13.41 | <0.0001 | -1.6442 | -1.1933 |  |  |  |
| Intercept | 1 | 0.4297 | 0.1191 | 3.61 | 0.0026 | 0.1758 | 0.6836 |  |  |  |
| Age |  | -0.00741 | 0.00201 | -3.69 | 0.0022 | -0.0117 | -0.00313 | 0.993 | 0.988 | 0.997 |
| Race/ethnicity | 1 | -0.3459 | 0.1264 | -2.74 | 0.0153 | -0.6154 | -0.0765 | 0.708 | 0.54 | 0.926 |
| Race/ethnicity | 2 | 0.0221 | 0.2461 | 0.09 | 0.9296 | -0.5024 | 0.5466 | 1.022 | 0.605 | 1.727 |
| Race/ethnicity | 4 | -0.0149 | 0.1159 | -0.13 | 0.8997 | -0.2618 | 0.2321 | 0.985 | 0.77 | 1.261 |
| Race/ethnicity | 5 | 0.3389 | 0.2249 | 1.51 | 0.1527 | -0.1405 | 0.8184 | 1.403 | 0.869 | 2.267 |
| Body mass index | 1 | 0.0798 | 0.2714 | 0.29 | 0.7728 | -0.4987 | 0.6583 | 1.083 | 0.607 | 1.931 |
| Body mass index | 3 | -0.0521 | 0.0865 | -0.6 | 0.5557 | -0.2365 | 0.1322 | 0.949 | 0.789 | 1.141 |
| Body mass index | 4 | 0.2259 | 0.1141 | 1.98 | 0.0662 | -0.0172 | 0.469 | 1.253 | 0.983 | 1.598 |
| Education | . | 1.0108 | 0.1204 | 8.39 | <0.0001 | 0.7541 | 1.2675 | 2.748 | 2.126 | 3.552 |
| Education | 11 | 0.1781 | 0.1021 | 1.74 | 0.1015 | -0.0395 | 0.3957 | 1.195 | 0.961 | 1.485 |
| Education | 12 | 0.0216 | 0.0983 | 0.22 | 0.8294 | -0.188 | 0.2311 | 1.022 | 0.829 | 1.26 |
| Exercise |  | -0.00591 | 0.00125 | -4.74 | 0.0003 | -0.00857 | -0.00326 | 0.994 | 0.991 | 0.997 |
| Cancer (y/n) | y | 0.3774 | 0.1697 | 2.22 | 0.0419 | 0.0157 | 0.739 | 1.458 | 1.016 | 2.094 |
| Retinyl stearate |  | -0.1464 | 0.0462 | -3.17 | 0.0063 | -0.2448 | -0.048 | 0.863812 | 0.782861 | 0.953134 |
|  |  |  |  |  |  |  |  |  |  |  |
| Parameter | | Estimate | Standard | t Value | p-value | 95% Confidence Limits | | Point Estimate | 95% Confidence Limits | |
|  |  |  | Error |  |  |  |  |  |  |  |
| Intercept | 3 | -2.3502 | 0.1722 | -13.65 | <0.0001 | -2.7172 | -1.9832 |  |  |  |
| Intercept | 2 | -1.5058 | 0.155 | -9.71 | <0.0001 | -1.8362 | -1.1754 |  |  |  |
| Intercept | 1 | 0.3445 | 0.1728 | 1.99 | 0.0648 | -0.0239 | 0.7129 |  |  |  |
| Age |  | -0.00785 | 0.00255 | -3.08 | 0.0077 | -0.0133 | -0.00241 | 0.992 | 0.987 | 0.998 |
| Race/ethnicity | 1 | -0.3265 | 0.1196 | -2.73 | 0.0155 | -0.5815 | -0.0715 | 0.721 | 0.559 | 0.931 |
| Race/ethnicity | 2 | 0.0223 | 0.2514 | 0.09 | 0.9304 | -0.5135 | 0.5582 | 1.023 | 0.598 | 1.748 |
| Race/ethnicity | 4 | -0.056 | 0.1158 | -0.48 | 0.6353 | -0.3028 | 0.1907 | 0.945 | 0.739 | 1.21 |
| Race/ethnicity | 5 | 0.3012 | 0.2044 | 1.47 | 0.1613 | -0.1345 | 0.7369 | 1.352 | 0.874 | 2.089 |
| Body mass index | 1 | 0.1285 | 0.2565 | 0.5 | 0.6235 | -0.4181 | 0.6752 | 1.137 | 0.658 | 1.964 |
| Body mass index | 3 | -0.0365 | 0.0864 | -0.42 | 0.6785 | -0.2207 | 0.1476 | 0.964 | 0.802 | 1.159 |
| Body mass index | 4 | 0.2557 | 0.1169 | 2.19 | 0.045 | 0.00646 | 0.5049 | 1.291 | 1.006 | 1.657 |
| Education | . | 1.0207 | 0.1123 | 9.09 | <0.0001 | 0.7814 | 1.2601 | 2.775 | 2.184 | 3.526 |
| Education | 11 | 0.1547 | 0.1106 | 1.4 | 0.1825 | -0.0812 | 0.3905 | 1.167 | 0.922 | 1.478 |
| Education | 12 | 0.00693 | 0.0964 | 0.07 | 0.9437 | -0.1986 | 0.2124 | 1.007 | 0.82 | 1.237 |
| Exercise |  | -0.00598 | 0.00123 | -4.86 | 0.0002 | -0.0086 | -0.00335 | 0.994 | 0.991 | 0.997 |
| Cancer (y/n) | y | 0.3654 | 0.1563 | 2.34 | 0.0337 | 0.0322 | 0.6986 | 1.441 | 1.033 | 2.011 |
| Vitamin A |  | -0.00004 | 0.00231 | -0.02 | 0.9855 | -0.00497 | 0.00488 | 0.99996 | 0.995042 | 1.004892 |
|  |  |  |  |  |  |  |  |  |  |  |
| Parameter | | Estimate | Standard | t Value | p-value | 95% Confidence Limits | | Point Estimate | 95% Confidence Limits | |
|  |  |  | Error |  |  |  |  |  |  |  |
| Intercept | 3 | -2.3651 | 0.1537 | -15.39 | <0.0001 | -2.6927 | -2.0376 |  |  |  |
| Intercept | 2 | -1.5208 | 0.1298 | -11.72 | <0.0001 | -1.7974 | -1.2442 |  |  |  |
| Intercept | 1 | 0.3295 | 0.1387 | 2.38 | 0.0313 | 0.0339 | 0.6252 |  |  |  |
| Age |  | -0.00803 | 0.00254 | -3.16 | 0.0064 | -0.0134 | -0.00262 | 0.992 | 0.987 | 0.997 |
| Race/ethnicity | 1 | -0.3267 | 0.1214 | -2.69 | 0.0168 | -0.5855 | -0.0679 | 0.721 | 0.557 | 0.934 |
| Race/ethnicity | 2 | 0.0226 | 0.2581 | 0.09 | 0.9315 | -0.5276 | 0.5727 | 1.023 | 0.59 | 1.773 |
| Race/ethnicity | 4 | -0.0527 | 0.1176 | -0.45 | 0.6604 | -0.3034 | 0.198 | 0.949 | 0.738 | 1.219 |
| Race/ethnicity | 5 | 0.3022 | 0.2056 | 1.47 | 0.1623 | -0.136 | 0.7404 | 1.353 | 0.873 | 2.097 |
| Body mass index | 1 | 0.1305 | 0.2578 | 0.51 | 0.62 | -0.419 | 0.6801 | 1.139 | 0.658 | 1.974 |
| Body mass index | 3 | -0.0384 | 0.0861 | -0.45 | 0.6619 | -0.2219 | 0.1451 | 0.962 | 0.801 | 1.156 |
| Body mass index | 4 | 0.2545 | 0.1153 | 2.21 | 0.0432 | 0.00883 | 0.5002 | 1.29 | 1.009 | 1.649 |
| Education | . | 1.0288 | 0.1058 | 9.73 | <0.0001 | 0.8033 | 1.2542 | 2.798 | 2.233 | 3.505 |
| Education | 11 | 0.1574 | 0.1132 | 1.39 | 0.1849 | -0.084 | 0.3987 | 1.17 | 0.919 | 1.49 |
| Education | 12 | 0.00827 | 0.098 | 0.08 | 0.9338 | -0.2005 | 0.2171 | 1.008 | 0.818 | 1.242 |
| Exercise |  | -0.00599 | 0.00123 | -4.87 | 0.0002 | -0.00861 | -0.00337 | 0.994 | 0.991 | 0.997 |
| Cancer (y/n) | y | 0.3648 | 0.1571 | 2.32 | 0.0348 | 0.0299 | 0.6997 | 1.44 | 1.03 | 2.013 |
| Vitamin E |  | 0.000016 | 0.000073 | 0.22 | 0.8276 | -0.00014 | 0.000172 | 1.000016 | 0.99986 | 1.000172 |
|  |  |  |  |  |  |  |  |  |  |  |
| Parameter | | Estimate | Standard | t Value | p-value | 95% Confidence Limits | | Point Estimate | 95% Confidence Limits | |
|  |  |  | Error |  |  |  |  |  |  |  |
| Intercept | 3 | -2.0953 | 0.1966 | -10.66 | <0.0001 | -2.5144 | -1.6762 |  |  |  |
| Intercept | 2 | -1.2446 | 0.1949 | -6.39 | <0.0001 | -1.6599 | -0.8292 |  |  |  |
| Intercept | 1 | 0.6063 | 0.2041 | 2.97 | 0.0095 | 0.1712 | 1.0414 |  |  |  |
| Age |  | -0.00891 | 0.00261 | -3.42 | 0.0038 | -0.0145 | -0.00336 | 0.991 | 0.986 | 0.997 |
| Race/ethnicity | 1 | -0.364 | 0.1187 | -3.07 | 0.0078 | -0.617 | -0.111 | 0.695 | 0.54 | 0.895 |
| Race/ethnicity | 2 | 0.0379 | 0.2516 | 0.15 | 0.8823 | -0.4984 | 0.5741 | 1.039 | 0.608 | 1.776 |
| Race/ethnicity | 4 | -0.0594 | 0.1238 | -0.48 | 0.6381 | -0.3232 | 0.2044 | 0.942 | 0.724 | 1.227 |
| Race/ethnicity | 5 | 0.2821 | 0.2006 | 1.41 | 0.18 | -0.1454 | 0.7097 | 1.326 | 0.865 | 2.033 |
| Body mass index | 1 | 0.0973 | 0.2654 | 0.37 | 0.7191 | -0.4685 | 0.6631 | 1.102 | 0.626 | 1.941 |
| Body mass index | 3 | -0.0274 | 0.0871 | -0.31 | 0.7574 | -0.2129 | 0.1582 | 0.973 | 0.808 | 1.171 |
| Body mass index | 4 | 0.2496 | 0.1179 | 2.12 | 0.0515 | -0.00183 | 0.5009 | 1.283 | 0.998 | 1.65 |
| Education | . | 0.9068 | 0.1534 | 5.91 | <0.0001 | 0.5799 | 1.2336 | 2.476 | 1.786 | 3.434 |
| Education | 11 | 0.107 | 0.1203 | 0.89 | 0.3877 | -0.1493 | 0.3633 | 1.113 | 0.861 | 1.438 |
| Education | 12 | 0.00339 | 0.0927 | 0.04 | 0.9713 | -0.1941 | 0.2009 | 1.003 | 0.824 | 1.223 |
| Exercise |  | -0.00582 | 0.00106 | -5.48 | <0.0001 | -0.00809 | -0.00356 | 0.994 | 0.992 | 0.996 |
| Cancer (y/n) | y | 0.3645 | 0.1535 | 2.37 | 0.0314 | 0.0373 | 0.6917 | 1.44 | 1.038 | 1.997 |
| Total lycopene |  | -0.00437 | 0.00226 | -1.93 | 0.0721 | -0.00919 | 0.000444 | 0.99564 | 0.990852 | 1.000444 |

## **Suppl. Table 2.** Results of adjusted model with the nutrient×cancer interaction term to explore the associations between carotenoids and cancer-related fatigue: all model output.

| Parameter | | Estimate | Standard | t Value | p-value | 95% Confidence Limits | | Odds Ratio Estimates | | |
| --- | --- | --- | --- | --- | --- | --- | --- | --- | --- | --- |
|  |  |  | Error |  |  |  |  | Point Estimate | 95% Confidence interval | |
| Intercept | 3 | -2.3359 | 0.1441 | -16.21 | <0.0001 | -2.6429 | -2.0288 |  |  |  |
| Intercept | 2 | -1.4908 | 0.1175 | -12.69 | <0.0001 | -1.7411 | -1.2404 |  |  |  |
| Intercept | 1 | 0.3586 | 0.1276 | 2.81 | 0.0132 | 0.0865 | 0.6307 |  |  |  |
| Age |  | -0.00761 | 0.0023 | -3.31 | 0.0047 | -0.0125 | -0.00272 | 0.992 | 0.988 | 0.997 |
| Race/ethnicity | 1 | -0.3193 | 0.121 | -2.64 | 0.0186 | -0.5772 | -0.0614 | 0.727 | 0.561 | 0.940 |
| Race/ethnicity | 2 | 0.0392 | 0.2632 | 0.15 | 0.8837 | -0.5218 | 0.6001 | 1.040 | 0.593 | 1.822 |
| Race/ethnicity | 4 | -0.055 | 0.1187 | -0.46 | 0.65 | -0.3079 | 0.198 | 0.947 | 0.735 | 1.219 |
| Race/ethnicity | 5 | 0.2993 | 0.2078 | 1.44 | 0.1704 | -0.1437 | 0.7423 | 1.349 | 0.866 | 2.101 |
| Body mass index | 1 | 0.1396 | 0.2614 | 0.53 | 0.6011 | -0.4175 | 0.6967 | 1.150 | 0.659 | 2.007 |
| Body mass index | 3 | -0.0483 | 0.0861 | -0.56 | 0.5834 | -0.2317 | 0.1352 | 0.953 | 0.793 | 1.145 |
| Body mass index | 4 | 0.2402 | 0.1156 | 2.08 | 0.0554 | -0.00632 | 0.4867 | 1.271 | 0.994 | 1.627 |
| Education | . | 1.0042 | 0.0966 | 10.4 | <0.0001 | 0.7983 | 1.21 | 2.730 | 2.222 | 3.354 |
| Education | 11 | 0.1387 | 0.1142 | 1.22 | 0.2431 | -0.1046 | 0.3821 | 1.149 | 0.901 | 1.465 |
| Education | 12 | -0.00428 | 0.0962 | -0.04 | 0.9651 | -0.2093 | 0.2007 | 0.996 | 0.811 | 1.222 |
| Exercise |  | -0.00592 | 0.00119 | -4.97 | 0.0002 | -0.00846 | -0.00338 | 0.994 | 0.992 | 0.997 |
| Cancer (y/n) | y | 0.5241 | 0.1794 | 2.92 | 0.0105 | 0.1418 | 0.9063 | 1.689 | 1.152 | 2.475 |
| α-Carotene |  | -0.00236 | 0.00504 | -0.47 | 0.6466 | -0.0131 | 0.00838 | 0.998 | 0.987 | 1.008 |
| α-Carotene*Cancer (y/n) | y | -0.0285 | 0.0194 | -1.47 | 0.1614 | -0.0698 | 0.0127 | 0.972 | 0.933 | 1.013 |
|  |  |  |  |  |  |  |  |  |  |  |
| Parameter | | Estimate | Standard | t Value | p-value | 95% Confidence Limits | | Point Estimate | 95% Confidence interval | |
|  |  |  | Error |  |  |  |  |  |  |  |
| Intercept | 3 | -2.3566 | 0.1476 | -15.97 | <0.0001 | -2.6711 | -2.0421 |  |  |  |
| Intercept | 2 | -1.5122 | 0.1246 | -12.14 | <0.0001 | -1.7777 | -1.2466 |  |  |  |
| Intercept | 1 | 0.3384 | 0.1378 | 2.46 | 0.0267 | 0.0447 | 0.6321 |  |  |  |
| Age |  | -0.00789 | 0.00222 | -3.55 | 0.0029 | -0.0126 | -0.00315 | 0.992 | 0.987 | 0.997 |
| Race/ethnicity | 1 | -0.3256 | 0.1219 | -2.67 | 0.0174 | -0.5854 | -0.0658 | 0.722 | 0.557 | 0.936 |
| Race/ethnicity | 2 | 0.0244 | 0.2588 | 0.09 | 0.9262 | -0.5272 | 0.576 | 1.025 | 0.590 | 1.779 |
| Race/ethnicity | 4 | -0.0547 | 0.1184 | -0.46 | 0.6506 | -0.3072 | 0.1977 | 0.947 | 0.736 | 1.219 |
| Race/ethnicity | 5 | 0.2981 | 0.2122 | 1.4 | 0.1805 | -0.1543 | 0.7505 | 1.347 | 0.857 | 2.118 |
| Body mass index | 1 | 0.1301 | 0.2556 | 0.51 | 0.6181 | -0.4147 | 0.6749 | 1.139 | 0.661 | 1.964 |
| Body mass index | 3 | -0.0374 | 0.0887 | -0.42 | 0.6797 | -0.2265 | 0.1518 | 0.963 | 0.797 | 1.164 |
| Body mass index | 4 | 0.2553 | 0.1146 | 2.23 | 0.0417 | 0.011 | 0.4996 | 1.291 | 1.011 | 1.648 |
| Education | . | 1.025 | 0.1026 | 9.99 | <0.0001 | 0.8064 | 1.2437 | 2.787 | 2.240 | 3.468 |
| Education | 11 | 0.1543 | 0.124 | 1.24 | 0.2326 | -0.1101 | 0.4187 | 1.167 | 0.896 | 1.520 |
| Education | 12 | 0.0072 | 0.103 | 0.07 | 0.9452 | -0.2124 | 0.2268 | 1.007 | 0.809 | 1.255 |
| Exercise |  | -0.00598 | 0.00122 | -4.91 | 0.0002 | -0.00858 | -0.00339 | 0.994 | 0.991 | 0.997 |
| Cancer (y/n) | y | 0.4362 | 0.2038 | 2.14 | 0.0491 | 0.00188 | 0.8705 | 1.547 | 1.002 | 2.388 |
| trans-β-Carotene |  | 0.000294 | 0.00246 | 0.12 | 0.9066 | -0.00496 | 0.00554 | 1.000 | 0.995 | 1.006 |
| trans-β-Carotene*Cancer (y/n) | y | -0.00295 | 0.00567 | -0.52 | 0.6109 | -0.015 | 0.00914 | 0.997 | 0.985 | 1.009 |
|  |  |  |  |  |  |  |  |  |  |  |
| Parameter | | Estimate | Standard | t Value | p-value | 95% Confidence Limits | | Point Estimate | 95% Confidence interval | |
|  |  |  | Error |  |  |  |  |  |  |  |
| Intercept | 3 | -2.4035 | 0.1485 | -16.19 | <0.0001 | -2.72 | -2.087 |  |  |  |
| Intercept | 2 | -1.5422 | 0.1453 | -10.62 | <0.0001 | -1.8519 | -1.2326 |  |  |  |
| Intercept | 1 | 0.3118 | 0.1649 | 1.89 | 0.0781 | -0.0397 | 0.6632 |  |  |  |
| Age |  | -0.00786 | 0.00234 | -3.36 | 0.0043 | -0.0128 | -0.00288 | 0.992 | 0.987 | 0.997 |
| Race/ethnicity | 1 | -0.3241 | 0.1409 | -2.3 | 0.0362 | -0.6244 | -0.0238 | 0.723 | 0.536 | 0.976 |
| Race/ethnicity | 2 | -0.00863 | 0.2756 | -0.03 | 0.9754 | -0.5961 | 0.5788 | 0.991 | 0.551 | 1.784 |
| Race/ethnicity | 4 | -0.0992 | 0.1214 | -0.82 | 0.4269 | -0.358 | 0.1596 | 0.906 | 0.699 | 1.173 |
| Race/ethnicity | 5 | 0.2888 | 0.1827 | 1.58 | 0.1348 | -0.1006 | 0.6783 | 1.335 | 0.904 | 1.971 |
| Body mass index | 1 | 0.0784 | 0.2589 | 0.3 | 0.7663 | -0.4735 | 0.6303 | 1.082 | 0.623 | 1.878 |
| Body mass index | 3 | -0.0183 | 0.0922 | -0.2 | 0.8454 | -0.2148 | 0.1782 | 0.982 | 0.807 | 1.195 |
| Body mass index | 4 | 0.2744 | 0.124 | 2.21 | 0.0428 | 0.0101 | 0.5386 | 1.316 | 1.010 | 1.714 |
| Education | . | 1.0442 | 0.1215 | 8.59 | <0.0001 | 0.7851 | 1.3032 | 2.841 | 2.193 | 3.681 |
| Education | 11 | 0.1808 | 0.1251 | 1.44 | 0.1691 | -0.0859 | 0.4475 | 1.198 | 0.918 | 1.564 |
| Education | 12 | 0.0654 | 0.1113 | 0.59 | 0.5656 | -0.1718 | 0.3025 | 1.068 | 0.842 | 1.353 |
| Exercise |  | -0.00614 | 0.00129 | -4.75 | 0.0003 | -0.0089 | -0.00338 | 0.994 | 0.991 | 0.997 |
| Cancer (y/n) | y | 0.4344 | 0.2075 | 2.09 | 0.0537 | -0.00788 | 0.8767 | 1.544 | 0.992 | 2.403 |
| cis-β-Carotene |  | 0.00505 | 0.0468 | 0.11 | 0.9155 | -0.0948 | 0.1049 | 1.005 | 0.910 | 1.111 |
| cis-β-Carotene*Cancer (y/n) | y | -0.0272 | 0.0892 | -0.3 | 0.7649 | -0.2173 | 0.163 | 0.973 | 0.805 | 1.177 |
|  |  |  |  |  |  |  |  |  |  |  |
| Parameter | | Estimate | Standard | t Value | p-value | 95% Confidence Limits | | Point Estimate | 95% Confidence interval | |
|  |  |  | Error |  |  |  |  |  |  |  |
| Intercept | 3 | -2.3833 | 0.1487 | -16.03 | <0.0001 | -2.7002 | -2.0664 |  |  |  |
| Intercept | 2 | -1.5368 | 0.121 | -12.7 | <0.0001 | -1.7947 | -1.279 |  |  |  |
| Intercept | 1 | 0.3131 | 0.1294 | 2.42 | 0.0288 | 0.0372 | 0.5889 |  |  |  |
| Age |  | -0.00786 | 0.00234 | -3.36 | 0.0043 | -0.0128 | -0.00287 | 0.992 | 0.987 | 0.997 |
| Race/ethnicity | 1 | -0.3587 | 0.123 | -2.92 | 0.0107 | -0.6209 | -0.0964 | 0.699 | 0.537 | 0.908 |
| Race/ethnicity | 2 | 0.0127 | 0.2555 | 0.05 | 0.961 | -0.5319 | 0.5573 | 1.013 | 0.587 | 1.746 |
| Race/ethnicity | 4 | -0.0615 | 0.118 | -0.52 | 0.6102 | -0.3131 | 0.1901 | 0.940 | 0.731 | 1.209 |
| Race/ethnicity | 5 | 0.2792 | 0.203 | 1.38 | 0.1891 | -0.1534 | 0.7118 | 1.322 | 0.858 | 2.038 |
| Body mass index | 1 | 0.1245 | 0.2523 | 0.49 | 0.6288 | -0.4133 | 0.6624 | 1.133 | 0.661 | 1.939 |
| Body mass index | 3 | -0.0286 | 0.0854 | -0.33 | 0.7424 | -0.2105 | 0.1534 | 0.972 | 0.810 | 1.166 |
| Body mass index | 4 | 0.2608 | 0.1183 | 2.2 | 0.0436 | 0.00854 | 0.513 | 1.298 | 1.009 | 1.670 |
| Education | . | 1.0293 | 0.1151 | 8.95 | <0.0001 | 0.7841 | 1.2745 | 2.799 | 2.190 | 3.577 |
| Education | 11 | 0.1591 | 0.1104 | 1.44 | 0.1699 | -0.0761 | 0.3944 | 1.172 | 0.927 | 1.483 |
| Education | 12 | 0.0216 | 0.0896 | 0.24 | 0.8124 | -0.1693 | 0.2125 | 1.022 | 0.844 | 1.237 |
| Exercise |  | -0.00605 | 0.00114 | -5.3 | <0.0001 | -0.00849 | -0.00362 | 0.994 | 0.992 | 0.996 |
| Cancer (y/n) | y | 0.5476 | 0.218 | 2.51 | 0.024 | 0.0829 | 1.0123 | 1.729 | 1.086 | 2.752 |
| β-Cryptoxanthin |  | 0.00276 | 0.00264 | 1.05 | 0.3113 | -0.00286 | 0.00838 | 1.003 | 0.997 | 1.008 |
| β-Cryptoxanthin*Cancer (y/n) | y | -0.0205 | 0.026 | -0.79 | 0.4444 | -0.076 | 0.0351 | 0.980 | 0.927 | 1.036 |
|  |  |  |  |  |  |  |  |  |  |  |
| Parameter | | Estimate | Standard | t Value | p-value | 95% Confidence Limits | | Point Estimate | 95% Confidence interval | |
|  |  |  | Error |  |  |  |  |  |  |  |
| Intercept | 3 | -2.4424 | 0.1168 | -20.91 | <0.0001 | -2.6914 | -2.1935 |  |  |  |
| Intercept | 2 | -1.5932 | 0.0977 | -16.3 | <0.0001 | -1.8015 | -1.3849 |  |  |  |
| Intercept | 1 | 0.2605 | 0.099 | 2.63 | 0.0189 | 0.0495 | 0.4714 |  |  |  |
| Age |  | -0.00737 | 0.00227 | -3.25 | 0.0054 | -0.0122 | -0.00253 | 0.993 | 0.988 | 0.997 |
| Race/ethnicity | 1 | -0.3188 | 0.1209 | -2.64 | 0.0187 | -0.5766 | -0.0611 | 0.727 | 0.562 | 0.941 |
| Race/ethnicity | 2 | 0.0357 | 0.2574 | 0.14 | 0.8916 | -0.513 | 0.5843 | 1.036 | 0.599 | 1.794 |
| Race/ethnicity | 4 | -0.0491 | 0.1187 | -0.41 | 0.6851 | -0.302 | 0.2039 | 0.952 | 0.739 | 1.226 |
| Race/ethnicity | 5 | 0.2908 | 0.203 | 1.43 | 0.1726 | -0.142 | 0.7236 | 1.338 | 0.868 | 2.062 |
| Body mass index | 1 | 0.1248 | 0.2504 | 0.5 | 0.6253 | -0.4088 | 0.6585 | 1.133 | 0.664 | 1.932 |
| Body mass index | 3 | -0.065 | 0.0827 | -0.79 | 0.4438 | -0.2413 | 0.1112 | 0.937 | 0.786 | 1.118 |
| Body mass index | 4 | 0.2009 | 0.1152 | 1.74 | 0.1016 | -0.0446 | 0.4464 | 1.222 | 0.956 | 1.563 |
| Education | . | 1.0139 | 0.1003 | 10.11 | <0.0001 | 0.8001 | 1.2277 | 2.756 | 2.226 | 3.413 |
| Education | 11 | 0.1319 | 0.1145 | 1.15 | 0.2674 | -0.1122 | 0.376 | 1.141 | 0.894 | 1.457 |
| Education | 12 | -0.0112 | 0.0933 | -0.12 | 0.9062 | -0.21 | 0.1877 | 0.989 | 0.811 | 1.206 |
| Exercise |  | -0.00581 | 0.00119 | -4.87 | 0.0002 | -0.00835 | -0.00327 | 0.994 | 0.992 | 0.997 |
| Cancer (y/n) | y | 0.0675 | 0.1978 | 0.34 | 0.7378 | -0.3541 | 0.4891 | 1.070 | 0.702 | 1.631 |
| γ-Tocopherol |  | 0.000438 | 0.000277 | 1.58 | 0.1355 | -0.00015 | 0.00103 | 1.000 | 1.000 | 1.001 |
| γ-Tocopherol*Cancer (y/n) | y | 0.00154 | 0.00089 | 1.73 | 0.1043 | -0.00036 | 0.00344 | 1.002 | 1.000 | 1.003 |
|  |  |  |  |  |  |  |  |  |  |  |
|  |  | Estimate | Standard | t Value | p-value | 95% Confidence Limits | | Point Estimate | 95% Confidence interval | |
|  |  |  | Error |  |  |  |  |  |  |  |
| Intercept | 3 | -2.3296 | 0.1686 | -13.82 | <0.0001 | -2.689 | -1.9703 |  |  |  |
| Intercept | 2 | -1.4837 | 0.1424 | -10.42 | <0.0001 | -1.7871 | -1.1802 |  |  |  |
| Intercept | 1 | 0.3693 | 0.1478 | 2.5 | 0.0246 | 0.0543 | 0.6843 |  |  |  |
| Age |  | -0.00739 | 0.0024 | -3.08 | 0.0076 | -0.0125 | -0.00228 | 0.993 | 0.988 | 0.998 |
| Race/ethnicity | 1 | -0.3129 | 0.1238 | -2.53 | 0.0232 | -0.5767 | -0.0491 | 0.731 | 0.562 | 0.952 |
| Race/ethnicity | 2 | 0.0414 | 0.2571 | 0.16 | 0.8743 | -0.5067 | 0.5895 | 1.042 | 0.602 | 1.803 |
| Race/ethnicity | 4 | -0.0437 | 0.1185 | -0.37 | 0.7175 | -0.2962 | 0.2089 | 0.957 | 0.744 | 1.232 |
| Race/ethnicity | 5 | 0.3021 | 0.2055 | 1.47 | 0.1623 | -0.136 | 0.7401 | 1.353 | 0.873 | 2.096 |
| Body mass index | 1 | 0.182 | 0.2538 | 0.72 | 0.4843 | -0.3589 | 0.7229 | 1.200 | 0.698 | 2.060 |
| Body mass index | 3 | -0.0445 | 0.0885 | -0.5 | 0.6221 | -0.2331 | 0.1441 | 0.956 | 0.792 | 1.155 |
| Body mass index | 4 | 0.2365 | 0.121 | 1.96 | 0.0694 | -0.0213 | 0.4944 | 1.267 | 0.979 | 1.639 |
| Education | . | 0.9972 | 0.085 | 11.73 | <0.0001 | 0.816 | 1.1784 | 2.711 | 2.261 | 3.249 |
| Education | 11 | 0.1413 | 0.1177 | 1.2 | 0.2486 | -0.1096 | 0.3922 | 1.152 | 0.896 | 1.480 |
| Education | 12 | -0.00563 | 0.1002 | -0.06 | 0.956 | -0.2193 | 0.208 | 0.994 | 0.803 | 1.231 |
| Exercise |  | -0.00588 | 0.00122 | -4.82 | 0.0002 | -0.00849 | -0.00328 | 0.994 | 0.992 | 0.997 |
| Cancer (y/n) | y | 0.945 | 0.2827 | 3.34 | 0.0044 | 0.3425 | 1.5476 | 2.573 | 1.408 | 4.700 |
| Lutein and zeaxanthin |  | -0.00208 | 0.00413 | -0.5 | 0.6219 | -0.0109 | 0.00672 | 0.998 | 0.989 | 1.007 |
| Lutein and zeaxanthin*Cancer (y/n) | y | -0.0345 | 0.0152 | -2.28 | 0.038 | -0.0668 | -0.00219 | 0.966 | 0.935 | 0.998 |
|  |  |  |  |  |  |  |  |  |  |  |
| Parameter | | Estimate | Standard | t Value | p-value | 95% Confidence Limits | | Point Estimate | 95% Confidence interval | |
|  |  |  | Error |  |  |  |  |  |  |  |
| Intercept | 3 | -2.0781 | 0.1814 | -11.45 | <0.0001 | -2.4648 | -1.6914 |  |  |  |
| Intercept | 2 | -1.2321 | 0.175 | -7.04 | <0.0001 | -1.605 | -0.8592 |  |  |  |
| Intercept | 1 | 0.6215 | 0.1851 | 3.36 | 0.0043 | 0.227 | 1.016 |  |  |  |
| Age |  | -0.00918 | 0.00265 | -3.47 | 0.0035 | -0.0148 | -0.00354 | 0.991 | 0.985 | 0.996 |
| Race/ethnicity | 1 | -0.3543 | 0.118 | -3 | 0.0089 | -0.6058 | -0.1029 | 0.702 | 0.546 | 0.902 |
| Race/ethnicity | 2 | 0.0206 | 0.2598 | 0.08 | 0.9379 | -0.5333 | 0.5744 | 1.021 | 0.587 | 1.776 |
| Race/ethnicity | 4 | -0.0533 | 0.1192 | -0.45 | 0.6614 | -0.3073 | 0.2008 | 0.948 | 0.735 | 1.222 |
| Race/ethnicity | 5 | 0.2904 | 0.2049 | 1.42 | 0.1767 | -0.1463 | 0.7271 | 1.337 | 0.864 | 2.069 |
| Body mass index | 1 | 0.1029 | 0.2686 | 0.38 | 0.7071 | -0.4696 | 0.6753 | 1.108 | 0.625 | 1.965 |
| Body mass index | 3 | -0.0228 | 0.0894 | -0.25 | 0.8022 | -0.2134 | 0.1678 | 0.977 | 0.808 | 1.183 |
| Body mass index | 4 | 0.2568 | 0.1183 | 2.17 | 0.0463 | 0.00476 | 0.5089 | 1.293 | 1.005 | 1.663 |
| Education | . | 0.8956 | 0.1472 | 6.08 | <0.0001 | 0.5818 | 1.2095 | 2.449 | 1.789 | 3.352 |
| Education | 11 | 0.1223 | 0.1158 | 1.06 | 0.3077 | -0.1245 | 0.369 | 1.130 | 0.883 | 1.446 |
| Education | 12 | -0.00221 | 0.0967 | -0.02 | 0.9821 | -0.2084 | 0.204 | 0.998 | 0.812 | 1.226 |
| Exercise |  | -0.0059 | 0.00114 | -5.19 | 0.0001 | -0.00833 | -0.00348 | 0.994 | 0.992 | 0.997 |
| Cancer (y/n) | y | 0.53 | 0.2499 | 2.12 | 0.051 | -0.00268 | 1.0627 | 1.699 | 0.997 | 2.894 |
| trans-Lycopene |  | -0.00859 | 0.0035 | -2.45 | 0.027 | -0.0161 | -0.00112 | 0.991 | 0.984 | 0.999 |
| trans-Lycopene*Cancer (y/n) | y | -0.00812 | 0.0149 | -0.54 | 0.594 | -0.0399 | 0.0237 | 0.992 | 0.961 | 1.024 |
|  |  |  |  |  |  |  |  |  |  |  |
| Parameter | | Estimate | Standard | t Value | p-value | 95% Confidence Limits | | Point Estimate | 95% Confidence interval | |
|  |  |  | Error |  |  |  |  |  |  |  |
| Intercept | 3 | -2.3166 | 0.1743 | -13.29 | <0.0001 | -2.688 | -1.9452 |  |  |  |
| Intercept | 2 | -1.4635 | 0.1459 | -10.03 | <0.0001 | -1.7746 | -1.1525 |  |  |  |
| Intercept | 1 | 0.3973 | 0.1559 | 2.55 | 0.0223 | 0.0649 | 0.7296 |  |  |  |
| Age |  | -0.00716 | 0.00252 | -2.84 | 0.0123 | -0.0125 | -0.0018 | 0.993 | 0.988 | 0.998 |
| Race/ethnicity | 1 | -0.3024 | 0.1088 | -2.78 | 0.0141 | -0.5344 | -0.0704 | 0.739 | 0.586 | 0.932 |
| Race/ethnicity | 2 | 0.0239 | 0.2494 | 0.1 | 0.9249 | -0.5077 | 0.5555 | 1.024 | 0.602 | 1.743 |
| Race/ethnicity | 4 | -0.0566 | 0.1158 | -0.49 | 0.6321 | -0.3035 | 0.1903 | 0.945 | 0.738 | 1.210 |
| Race/ethnicity | 5 | 0.2832 | 0.2082 | 1.36 | 0.1938 | -0.1605 | 0.727 | 1.327 | 0.852 | 2.069 |
| Body mass index | 1 | 0.1136 | 0.2625 | 0.43 | 0.6712 | -0.4458 | 0.673 | 1.120 | 0.640 | 1.960 |
| Body mass index | 3 | -0.0125 | 0.0887 | -0.14 | 0.89 | -0.2016 | 0.1767 | 0.988 | 0.817 | 1.193 |
| Body mass index | 4 | 0.2649 | 0.1193 | 2.22 | 0.0423 | 0.0105 | 0.5192 | 1.303 | 1.011 | 1.681 |
| Education | . | 0.9458 | 0.11 | 8.6 | <0.0001 | 0.7114 | 1.1803 | 2.575 | 2.037 | 3.255 |
| Education | 11 | 0.126 | 0.1227 | 1.03 | 0.3206 | -0.1355 | 0.3876 | 1.134 | 0.873 | 1.473 |
| Education | 12 | -0.0143 | 0.1082 | -0.13 | 0.8963 | -0.245 | 0.2163 | 0.986 | 0.783 | 1.242 |
| Exercise |  | -0.00561 | 0.00121 | -4.65 | 0.0003 | -0.00818 | -0.00304 | 0.994 | 0.992 | 0.997 |
| Cancer (y/n) | y | 0.5176 | 0.277 | 1.87 | 0.0814 | -0.0729 | 1.1081 | 1.678 | 0.930 | 3.029 |
| Retinyl palmitate |  | -0.0309 | 0.0196 | -1.58 | 0.136 | -0.0726 | 0.0109 | 0.970 | 0.930 | 1.011 |
| Retinyl palmitate*Cancer (y/n) | y | -0.0787 | 0.087 | -0.9 | 0.3799 | -0.2642 | 0.1067 | 0.924 | 0.768 | 1.113 |
|  |  |  |  |  |  |  |  |  |  |  |
| Parameter | | Estimate | Standard | t Value | p-value | 95% Confidence Limits | | Point Estimate | 95% Confidence interval | |
|  |  |  | Error |  |  |  |  |  |  |  |
| Intercept | 3 | -2.2545 | 0.1344 | -16.78 | <0.0001 | -2.5409 | -1.9681 |  |  |  |
| Intercept | 2 | -1.4313 | 0.1106 | -12.94 | <0.0001 | -1.667 | -1.1956 |  |  |  |
| Intercept | 1 | 0.4173 | 0.1219 | 3.42 | 0.0038 | 0.1575 | 0.6771 |  |  |  |
| Age |  | -0.00745 | 0.00202 | -3.69 | 0.0022 | -0.0118 | -0.00314 | 0.993 | 0.988 | 0.997 |
| Race/ethnicity | 1 | -0.3449 | 0.1271 | -2.71 | 0.016 | -0.6158 | -0.074 | 0.708 | 0.540 | 0.929 |
| Race/ethnicity | 2 | 0.0275 | 0.2465 | 0.11 | 0.9127 | -0.498 | 0.553 | 1.028 | 0.608 | 1.738 |
| Race/ethnicity | 4 | -0.0142 | 0.1167 | -0.12 | 0.905 | -0.2629 | 0.2345 | 0.986 | 0.769 | 1.264 |
| Race/ethnicity | 5 | 0.3417 | 0.2246 | 1.52 | 0.1491 | -0.1371 | 0.8204 | 1.407 | 0.872 | 2.271 |
| Body mass index | 1 | 0.0862 | 0.2698 | 0.32 | 0.7537 | -0.4889 | 0.6613 | 1.090 | 0.613 | 1.937 |
| Body mass index | 3 | -0.0543 | 0.0853 | -0.64 | 0.5342 | -0.2361 | 0.1276 | 0.947 | 0.790 | 1.136 |
| Body mass index | 4 | 0.2241 | 0.1139 | 1.97 | 0.0679 | -0.0187 | 0.4669 | 1.251 | 0.982 | 1.595 |
| Education | . | 1.0144 | 0.1203 | 8.43 | <0.0001 | 0.7579 | 1.2709 | 2.758 | 2.134 | 3.564 |
| Education | 11 | 0.1798 | 0.1021 | 1.76 | 0.0986 | -0.0378 | 0.3975 | 1.197 | 0.963 | 1.488 |
| Education | 12 | 0.0227 | 0.0988 | 0.23 | 0.8211 | -0.1878 | 0.2333 | 1.023 | 0.829 | 1.263 |
| Exercise |  | -0.00592 | 0.00126 | -4.71 | 0.0003 | -0.0086 | -0.00325 | 0.994 | 0.991 | 0.997 |
| Cancer (y/n) | y | 0.5251 | 0.2938 | 1.79 | 0.0941 | -0.1012 | 1.1514 | 1.691 | 0.904 | 3.163 |
| Retinyl stearate |  | -0.1247 | 0.0641 | -1.95 | 0.0705 | -0.2613 | 0.0118 | 0.883 | 0.770 | 1.012 |
| Retinyl stearate*Cancer (y/n) | y | -0.2168 | 0.302 | -0.72 | 0.4837 | -0.8605 | 0.4268 | 0.805 | 0.423 | 1.532 |
|  |  |  |  |  |  |  |  |  |  |  |
| Parameter | | Estimate | Standard | t Value | p-value | 95% Confidence Limits | | Point Estimate | 95% Confidence interval | |
|  |  |  | Error |  |  |  |  |  |  |  |
| Intercept | 3 | -2.3273 | 0.1715 | -13.57 | <0.0001 | -2.6929 | -1.9617 |  |  |  |
| Intercept | 2 | -1.4828 | 0.1508 | -9.83 | <0.0001 | -1.8041 | -1.1614 |  |  |  |
| Intercept | 1 | 0.3679 | 0.1721 | 2.14 | 0.0494 | 0.00114 | 0.7346 |  |  |  |
| Age |  | -0.0078 | 0.00256 | -3.05 | 0.0081 | -0.0133 | -0.00235 | 0.992 | 0.987 | 0.998 |
| Race/ethnicity | 1 | -0.3286 | 0.1185 | -2.77 | 0.0142 | -0.5811 | -0.0761 | 0.720 | 0.559 | 0.927 |
| Race/ethnicity | 2 | 0.0217 | 0.2514 | 0.09 | 0.9325 | -0.5141 | 0.5574 | 1.022 | 0.598 | 1.746 |
| Race/ethnicity | 4 | -0.0591 | 0.1165 | -0.51 | 0.6192 | -0.3074 | 0.1892 | 0.943 | 0.735 | 1.208 |
| Race/ethnicity | 5 | 0.3011 | 0.205 | 1.47 | 0.1625 | -0.1358 | 0.738 | 1.351 | 0.873 | 2.092 |
| Body mass index | 1 | 0.1328 | 0.258 | 0.51 | 0.6142 | -0.4171 | 0.6827 | 1.142 | 0.659 | 1.979 |
| Body mass index | 3 | -0.0348 | 0.0867 | -0.4 | 0.6938 | -0.2195 | 0.1499 | 0.966 | 0.803 | 1.162 |
| Body mass index | 4 | 0.2571 | 0.117 | 2.2 | 0.0442 | 0.00762 | 0.5066 | 1.293 | 1.008 | 1.660 |
| Education | . | 1.0085 | 0.1113 | 9.06 | <0.0001 | 0.7714 | 1.2457 | 2.742 | 2.163 | 3.475 |
| Education | 11 | 0.1537 | 0.11 | 1.4 | 0.1824 | -0.0807 | 0.3881 | 1.166 | 0.923 | 1.474 |
| Education | 12 | 0.00632 | 0.0962 | 0.07 | 0.9485 | -0.1986 | 0.2113 | 1.006 | 0.820 | 1.235 |
| Exercise |  | -0.00595 | 0.00123 | -4.82 | 0.0002 | -0.00858 | -0.00332 | 0.994 | 0.991 | 0.997 |
| Cancer (y/n) | y | 0.0519 | 0.3721 | 0.14 | 0.891 | -0.7412 | 0.845 | 1.053 | 0.477 | 2.328 |
| Vitamin A |  | -0.00047 | 0.00246 | -0.19 | 0.8509 | -0.00572 | 0.00478 | 1.000 | 0.994 | 1.005 |
| Vitamin A*Cancer (y/n) | y | 0.00481 | 0.00619 | 0.78 | 0.4495 | -0.00839 | 0.018 | 1.005 | 0.992 | 1.018 |
|  |  |  |  |  |  |  |  |  |  |  |
| Parameter | | Estimate | Standard | t Value | p-value | 95% Confidence Limits | | Point Estimate | 95% Confidence interval | |
|  |  |  | Error |  |  |  |  |  |  |  |
| Intercept | 3 | -2.3568 | 0.1637 | -14.4 | <0.0001 | -2.7058 | -2.0079 |  |  |  |
| Intercept | 2 | -1.5124 | 0.1374 | -11 | <0.0001 | -1.8053 | -1.2195 |  |  |  |
| Intercept | 1 | 0.3382 | 0.1507 | 2.24 | 0.0403 | 0.017 | 0.6593 |  |  |  |
| Age |  | -0.00799 | 0.00256 | -3.12 | 0.0071 | -0.0135 | -0.00252 | 0.992 | 0.987 | 0.997 |
| Race/ethnicity | 1 | -0.3267 | 0.1214 | -2.69 | 0.0168 | -0.5855 | -0.0678 | 0.721 | 0.557 | 0.934 |
| Race/ethnicity | 2 | 0.0219 | 0.2584 | 0.08 | 0.9336 | -0.529 | 0.5727 | 1.022 | 0.589 | 1.773 |
| Race/ethnicity | 4 | -0.0533 | 0.1173 | -0.45 | 0.6561 | -0.3033 | 0.1967 | 0.948 | 0.738 | 1.217 |
| Race/ethnicity | 5 | 0.3037 | 0.2067 | 1.47 | 0.1624 | -0.1369 | 0.7442 | 1.355 | 0.872 | 2.105 |
| Body mass index | 1 | 0.1287 | 0.2619 | 0.49 | 0.6301 | -0.4294 | 0.6869 | 1.137 | 0.651 | 1.987 |
| Body mass index | 3 | -0.0376 | 0.0854 | -0.44 | 0.6663 | -0.2196 | 0.1445 | 0.963 | 0.803 | 1.155 |
| Body mass index | 4 | 0.2551 | 0.1146 | 2.23 | 0.0418 | 0.0108 | 0.4994 | 1.291 | 1.011 | 1.648 |
| Education | . | 1.026 | 0.1032 | 9.95 | <0.0001 | 0.8061 | 1.2459 | 2.790 | 2.239 | 3.476 |
| Education | 11 | 0.1574 | 0.1131 | 1.39 | 0.1843 | -0.0836 | 0.3984 | 1.170 | 0.920 | 1.489 |
| Education | 12 | 0.0078 | 0.0978 | 0.08 | 0.9375 | -0.2006 | 0.2162 | 1.008 | 0.818 | 1.241 |
| Exercise |  | -0.00598 | 0.00125 | -4.78 | 0.0002 | -0.00865 | -0.00332 | 0.994 | 0.991 | 0.997 |
| Cancer (y/n) | y | 0.2169 | 0.4153 | 0.52 | 0.6092 | -0.6684 | 1.1021 | 1.242 | 0.513 | 3.010 |
| Vitamin E |  | 7.58E-06 | 0.000097 | 0.08 | 0.9385 | -0.0002 | 0.000213 | 1.000 | 1.000 | 1.000 |
| Vitamin E*Cancer (y/n) | y | 0.000101 | 0.000332 | 0.31 | 0.764 | -0.00061 | 0.000809 | 1.000 | 0.999 | 1.001 |
|  |  |  |  |  |  |  |  |  |  |  |
| Parameter | | Estimate | Standard | t Value | p-value | 95% Confidence Limits | | Point Estimate | 95% Confidence interval | |
|  |  |  | Error |  |  |  |  |  |  |  |
| Intercept | 3 | -2.1048 | 0.1902 | -11.06 | <0.0001 | -2.5103 | -1.6994 |  |  |  |
| Intercept | 2 | -1.254 | 0.1858 | -6.75 | <0.0001 | -1.65 | -0.8581 |  |  |  |
| Intercept | 1 | 0.5969 | 0.1951 | 3.06 | 0.008 | 0.181 | 1.0128 |  |  |  |
| Age |  | -0.00892 | 0.00262 | -3.4 | 0.0039 | -0.0145 | -0.00333 | 0.991 | 0.986 | 0.997 |
| Race/ethnicity | 1 | -0.3635 | 0.1186 | -3.06 | 0.0079 | -0.6163 | -0.1107 | 0.695 | 0.540 | 0.895 |
| Race/ethnicity | 2 | 0.0382 | 0.2515 | 0.15 | 0.8813 | -0.4978 | 0.5742 | 1.039 | 0.608 | 1.776 |
| Race/ethnicity | 4 | -0.0594 | 0.1237 | -0.48 | 0.6377 | -0.3231 | 0.2042 | 0.942 | 0.724 | 1.226 |
| Race/ethnicity | 5 | 0.2829 | 0.2014 | 1.4 | 0.1804 | -0.1463 | 0.7122 | 1.327 | 0.864 | 2.038 |
| Body mass index | 1 | 0.1003 | 0.2686 | 0.37 | 0.7141 | -0.4723 | 0.6729 | 1.106 | 0.624 | 1.960 |
| Body mass index | 3 | -0.0262 | 0.0887 | -0.3 | 0.7719 | -0.2153 | 0.1629 | 0.974 | 0.806 | 1.177 |
| Body mass index | 4 | 0.2504 | 0.1188 | 2.11 | 0.0523 | -0.00287 | 0.5037 | 1.285 | 0.997 | 1.655 |
| Education | . | 0.9107 | 0.1491 | 6.11 | <0.0001 | 0.5929 | 1.2285 | 2.486 | 1.809 | 3.416 |
| Education | 11 | 0.107 | 0.1206 | 0.89 | 0.3889 | -0.15 | 0.364 | 1.113 | 0.861 | 1.439 |
| Education | 12 | 0.00301 | 0.093 | 0.03 | 0.9746 | -0.1953 | 0.2013 | 1.003 | 0.823 | 1.223 |
| Exercise |  | -0.00582 | 0.00105 | -5.53 | <0.0001 | -0.00806 | -0.00357 | 0.994 | 0.992 | 0.996 |
| Cancer (y/n) | y | 0.4825 | 0.2658 | 1.81 | 0.0896 | -0.0842 | 1.0491 | 1.620 | 0.919 | 2.855 |
| Total lycopene |  | -0.00417 | 0.0021 | -1.99 | 0.065 | -0.00864 | 0.000295 | 0.996 | 0.991 | 1.000 |
| Total lycopene*Cancer (y/n) | y | -0.00293 | 0.00858 | -0.34 | 0.7374 | -0.0212 | 0.0154 | 0.997 | 0.979 | 1.016 |

## **Suppl. Table 3.** Adjusted odds ratios and 95% confidence intervals for the effects of the nutrient×cancer interaction on fatigue. Models are adjusted for age, body mass index, race/ethnicity, education, physical activity, history of a cancer diagnosis, and nutrient concentration.

|  | **Odds Ratio** | **Lower 95% confidence limit** | **Upper 95% confidence limit** | **p-value** |
| --- | --- | --- | --- | --- |
| **α-Carotene** | 0.972 | 0.933 | 1.013 | 0.161 |
| ***trans*-β-Carotene** | 0.997 | 0.985 | 1.009 | 0.611 |
| ***cis*-β-Carotene** | 0.973 | 0.805 | 1.177 | 0.765 |
| **β-Cryptoxanthin** | 0.980 | 0.927 | 1.036 | 0.444 |
| **γ-Tocopherol** | 1.002 | 1.000 | 1.003 | 0.104 |
| **Lutein and zeaxanthin** | 0.966 | 0.935 | 0.998 | 0.038* |
| ***trans*-Lycopene** | 0.992 | 0.961 | 1.024 | 0.594 |
| **Retinyl palmitate** | 0.924 | 0.768 | 1.113 | 0.380 |
| **Retinyl stearate** | 0.805 | 0.423 | 1.532 | 0.484 |
| **Vitamin A** | 1.005 | 0.992 | 1.018 | 0.450 |
| **Vitamin E** | 1.000 | 0.999 | 1.001 | 0.764 |
| **Total (*cis*- and *trans*-) lycopene** | 0.997 | 0.979 | 1.016 | 0.737 |

## **Suppl. Table 4.** Results of adjusted models (no interaction term) describing the association between carotenoid concentrations and fatigue only among cancer survivors: all model output; estimates for race/ethnicity are compared to non-Hispanic Whites, 1=Mexican American, 2=Other Hispanic, 4=non-Hispanic Black American, 5=Other non-Hispanic race, including multi-racial; estimates for body mass index are compared to those of normal weight; and estimates for education are compared to those with at least a college education. Exercise is estimated from metabolic equivalents (MET hours) per week as a continuous variable. Age, Years since Diagnosis, and carotenoid concentration are treated as continuous variables. (n=272)

| **Parameter** |  | **Estimate** | **Standard**  **Error** | **t Value** | **p-value** | **95% Confidence Limits** | | **Odds Ratio Estimates** | | |
| --- | --- | --- | --- | --- | --- | --- | --- | --- | --- | --- |
|  |  |  |  |  |  |  |  | **Point Estimate** | **95% Confidence Limits** | |
| Intercept | 3 | -1.503 | 0.908 | -1.660 | 0.118 | -3.437 | 0.431 |  |  |  |
| Intercept | 2 | -0.776 | 0.870 | -0.890 | 0.387 | -2.629 | 1.078 |  |  |  |
| Intercept | 1 | 0.684 | 0.919 | 0.740 | 0.468 | -1.274 | 2.642 |  |  |  |
| Age |  | -0.018 | 0.011 | -1.620 | 0.126 | -0.041 | 0.006 | 0.982 | 0.960 | 1.006 |
| Race/ethnicity | 1 | 0.637 | 0.596 | 1.070 | 0.302 | -0.633 | 1.907 | 1.891 | 0.531 | 6.734 |
| Race/ethnicity | 2 | -1.165 | 1.631 | -0.710 | 0.486 | -4.641 | 2.310 | 0.312 | 0.010 | 10.076 |
| Race/ethnicity | 4 | -0.284 | 0.476 | -0.600 | 0.559 | -1.299 | 0.730 | 0.753 | 0.273 | 2.076 |
| Race/ethnicity | 5 | -0.520 | 1.011 | -0.510 | 0.614 | -2.675 | 1.635 | 0.594 | 0.069 | 5.127 |
| Body mass index | 1 | -0.582 | 1.134 | -0.510 | 0.615 | -3.000 | 1.835 | 0.559 | 0.050 | 6.266 |
| Body mass index | 3 | 0.164 | 0.365 | 0.450 | 0.660 | -0.613 | 0.941 | 1.178 | 0.542 | 2.562 |
| Body mass index | 4 | 0.306 | 0.295 | 1.040 | 0.315 | -0.321 | 0.934 | 1.359 | 0.725 | 2.545 |
| Education | 11 | 0.719 | 0.204 | 3.530 | 0.003 | 0.285 | 1.153 | 2.053 | 1.330 | 3.167 |
| Education | 12 | 0.466 | 0.295 | 1.580 | 0.134 | -0.162 | 1.094 | 1.594 | 0.851 | 2.986 |
| Exercise |  | -0.001 | 0.003 | -0.250 | 0.805 | -0.008 | 0.006 | 0.999 | 0.992 | 1.006 |
| Years since diagnosis |  | 0.009 | 0.011 | 0.860 | 0.402 | -0.014 | 0.033 | 1.009 | 0.986 | 1.033 |
| α-Carotene |  | -0.013 | 0.016 | -0.780 | 0.450 | -0.048 | 0.022 | 0.987 | 0.953 | 1.022 |
|  |  |  |  |  |  |  |  |  |  |  |
| Parameter | | Estimate | Standard Error | t Value | p-value | 95% Confidence Limits | | Point Estimate | 95% Confidence Limits | |
| Intercept | 3 | -1.613 | 0.917 | -1.760 | 0.099 | -3.568 | 0.343 |  |  |  |
| Intercept | 2 | -0.886 | 0.885 | -1.000 | 0.332 | -2.772 | 0.999 |  |  |  |
| Intercept | 1 | 0.573 | 0.936 | 0.610 | 0.550 | -1.421 | 2.567 |  |  |  |
| Age |  | -0.018 | 0.011 | -1.710 | 0.107 | -0.041 | 0.004 | 0.982 | 0.960 | 1.004 |
| Race/ethnicity | 1 | 0.619 | 0.583 | 1.060 | 0.306 | -0.625 | 1.862 | 1.857 | 0.535 | 6.439 |
| Race/ethnicity | 2 | -1.224 | 1.606 | -0.760 | 0.458 | -4.648 | 2.199 | 0.294 | 0.010 | 9.014 |
| Race/ethnicity | 4 | -0.282 | 0.462 | -0.610 | 0.550 | -1.266 | 0.702 | 0.754 | 0.282 | 2.017 |
| Race/ethnicity | 5 | -0.518 | 1.007 | -0.510 | 0.615 | -2.664 | 1.629 | 0.596 | 0.070 | 5.097 |
| Body mass index | 1 | -0.606 | 1.086 | -0.560 | 0.585 | -2.920 | 1.708 | 0.545 | 0.054 | 5.516 |
| Body mass index | 3 | 0.214 | 0.346 | 0.620 | 0.546 | -0.523 | 0.950 | 1.238 | 0.593 | 2.586 |
| Body mass index | 4 | 0.375 | 0.301 | 1.240 | 0.233 | -0.267 | 1.018 | 1.455 | 0.765 | 2.766 |
| Education | 11 | 0.771 | 0.221 | 3.490 | 0.003 | 0.300 | 1.242 | 2.161 | 1.350 | 3.462 |
| Education | 12 | 0.507 | 0.308 | 1.640 | 0.121 | -0.150 | 1.164 | 1.660 | 0.860 | 3.202 |
| Exercise |  | -0.001 | 0.003 | -0.260 | 0.795 | -0.008 | 0.006 | 0.999 | 0.992 | 1.006 |
| Years since diagnosis |  | 0.009 | 0.011 | 0.830 | 0.420 | -0.014 | 0.032 | 1.009 | 0.986 | 1.033 |
| trans-β-Carotene |  | 0.000 | 0.005 | 0.090 | 0.929 | -0.010 | 0.011 | 1.000 | 0.990 | 1.011 |
|  |  |  |  |  |  |  |  |  |  |  |
| Parameter | | Estimate | Standard Error | t Value | p-value | 95% Confidence Limits |  | Point Estimate | 95% Confidence Limits |  |
| Intercept | 3 | -1.356 | 0.946 | -1.430 | 0.172 | -3.372 | 0.659 |  |  |  |
| Intercept | 2 | -0.635 | 0.930 | -0.680 | 0.505 | -2.617 | 1.347 |  |  |  |
| Intercept | 1 | 0.848 | 0.981 | 0.860 | 0.401 | -1.242 | 2.938 |  |  |  |
| Age |  | -0.023 | 0.011 | -2.080 | 0.055 | -0.047 | 0.001 | 0.977 | 0.954 | 1.001 |
| Race/ethnicity | 1 | 0.500 | 0.653 | 0.770 | 0.456 | -0.891 | 1.890 | 1.648 | 0.410 | 6.621 |
| Race/ethnicity | 2 | -0.883 | 1.551 | -0.570 | 0.578 | -4.190 | 2.424 | 0.413 | 0.015 | 11.288 |
| Race/ethnicity | 4 | -0.332 | 0.465 | -0.710 | 0.486 | -1.323 | 0.659 | 0.717 | 0.266 | 1.933 |
| Race/ethnicity | 5 | -0.644 | 0.976 | -0.660 | 0.520 | -2.723 | 1.436 | 0.525 | 0.066 | 4.206 |
| Body mass index | 1 | -0.640 | 1.084 | -0.590 | 0.564 | -2.949 | 1.670 | 0.527 | 0.052 | 5.310 |
| Body mass index | 3 | 0.271 | 0.334 | 0.810 | 0.430 | -0.440 | 0.981 | 1.311 | 0.644 | 2.668 |
| Body mass index | 4 | 0.376 | 0.341 | 1.100 | 0.287 | -0.351 | 1.104 | 1.457 | 0.704 | 3.016 |
| Education | 11 | 0.769 | 0.231 | 3.330 | 0.005 | 0.277 | 1.261 | 2.158 | 1.319 | 3.530 |
| Education | 12 | 0.505 | 0.271 | 1.860 | 0.082 | -0.073 | 1.084 | 1.657 | 0.930 | 2.955 |
| Exercise |  | -0.002 | 0.004 | -0.410 | 0.685 | -0.010 | 0.006 | 0.998 | 0.991 | 1.006 |
| Years since diagnosis |  | 0.011 | 0.011 | 1.030 | 0.319 | -0.012 | 0.034 | 1.011 | 0.988 | 1.034 |
| cis-β-Carotene |  | 0.030 | 0.071 | 0.420 | 0.681 | -0.122 | 0.182 | 1.030 | 0.885 | 1.199 |
|  |  |  |  |  |  |  |  |  |  |  |
| Parameter | | Estimate | Standard Error | t Value | p-value | 95% Confidence Limits |  | Point Estimate | 95% Confidence Limits |  |
| Intercept | 3 | -1.572 | 0.857 | -1.830 | 0.087 | -3.400 | 0.255 |  |  |  |
| Intercept | 2 | -0.846 | 0.822 | -1.030 | 0.320 | -2.598 | 0.907 |  |  |  |
| Intercept | 1 | 0.620 | 0.868 | 0.710 | 0.486 | -1.230 | 2.470 |  |  |  |
| Age |  | -0.018 | 0.012 | -1.520 | 0.150 | -0.042 | 0.007 | 0.983 | 0.959 | 1.007 |
| Race/ethnicity | 1 | 0.663 | 0.606 | 1.090 | 0.291 | -0.629 | 1.954 | 1.940 | 0.533 | 7.058 |
| Race/ethnicity | 2 | -1.191 | 1.661 | -0.720 | 0.484 | -4.732 | 2.350 | 0.304 | 0.009 | 10.486 |
| Race/ethnicity | 4 | -0.271 | 0.471 | -0.580 | 0.574 | -1.276 | 0.733 | 0.762 | 0.279 | 2.082 |
| Race/ethnicity | 5 | -0.559 | 0.996 | -0.560 | 0.583 | -2.683 | 1.565 | 0.572 | 0.068 | 4.782 |
| Body mass index | 1 | -0.618 | 1.086 | -0.570 | 0.578 | -2.933 | 1.697 | 0.539 | 0.053 | 5.459 |
| Body mass index | 3 | 0.239 | 0.334 | 0.720 | 0.485 | -0.473 | 0.952 | 1.270 | 0.623 | 2.590 |
| Body mass index | 4 | 0.351 | 0.304 | 1.150 | 0.266 | -0.297 | 0.999 | 1.421 | 0.743 | 2.717 |
| Education | 11 | 0.736 | 0.231 | 3.180 | 0.006 | 0.243 | 1.229 | 2.088 | 1.275 | 3.418 |
| Education | 12 | 0.516 | 0.277 | 1.860 | 0.082 | -0.074 | 1.106 | 1.675 | 0.929 | 3.023 |
| Exercise |  | -0.001 | 0.003 | -0.250 | 0.803 | -0.008 | 0.006 | 0.999 | 0.992 | 1.007 |
| Years since diagnosis |  | 0.008 | 0.011 | 0.740 | 0.470 | -0.015 | 0.031 | 1.008 | 0.985 | 1.031 |
| β-Cryptoxanthin |  | -0.007 | 0.025 | -0.290 | 0.776 | -0.060 | 0.046 | 0.993 | 0.942 | 1.047 |
|  |  |  |  |  |  |  |  |  |  |  |
| Parameter | | Estimate | Standard Error | t Value | p-value | 95% Confidence Limits |  | Point Estimate | 95% Confidence Limits |  |
| Intercept | 3 | -1.827 | 0.840 | -2.180 | 0.046 | -3.617 | -0.037 |  |  |  |
| Intercept | 2 | -1.097 | 0.806 | -1.360 | 0.194 | -2.814 | 0.621 |  |  |  |
| Intercept | 1 | 0.372 | 0.855 | 0.430 | 0.670 | -1.451 | 2.194 |  |  |  |
| Age |  | -0.017 | 0.011 | -1.580 | 0.136 | -0.041 | 0.006 | 0.983 | 0.960 | 1.006 |
| Race/ethnicity | 1 | 0.627 | 0.568 | 1.100 | 0.287 | -0.584 | 1.839 | 1.872 | 0.557 | 6.288 |
| Race/ethnicity | 2 | -1.176 | 1.532 | -0.770 | 0.455 | -4.441 | 2.089 | 0.309 | 0.012 | 8.078 |
| Race/ethnicity | 4 | -0.304 | 0.480 | -0.630 | 0.536 | -1.328 | 0.720 | 0.738 | 0.265 | 2.054 |
| Race/ethnicity | 5 | -0.618 | 0.991 | -0.620 | 0.542 | -2.730 | 1.493 | 0.539 | 0.065 | 4.450 |
| Body mass index | 1 | -0.687 | 1.071 | -0.640 | 0.531 | -2.969 | 1.595 | 0.503 | 0.051 | 4.926 |
| Body mass index | 3 | 0.147 | 0.374 | 0.390 | 0.701 | -0.651 | 0.944 | 1.158 | 0.521 | 2.571 |
| Body mass index | 4 | 0.244 | 0.331 | 0.740 | 0.472 | -0.461 | 0.950 | 1.277 | 0.630 | 2.585 |
| Education | 11 | 0.645 | 0.229 | 2.820 | 0.013 | 0.157 | 1.132 | 1.905 | 1.170 | 3.102 |
| Education | 12 | 0.450 | 0.313 | 1.440 | 0.171 | -0.217 | 1.117 | 1.568 | 0.805 | 3.056 |
| Exercise |  | -0.001 | 0.004 | -0.240 | 0.813 | -0.008 | 0.007 | 0.999 | 0.992 | 1.007 |
| Years since diagnosis |  | 0.010 | 0.011 | 0.920 | 0.374 | -0.013 | 0.033 | 1.010 | 0.987 | 1.033 |
| γ-Tocopherol |  | 0.001 | 0.001 | 1.410 | 0.179 | -0.001 | 0.003 | 1.001 | 0.999 | 1.003 |
|  |  |  |  |  |  |  |  |  |  |  |
| Parameter | | Estimate | Standard Error | t Value | p-value | 95% Confidence Limits |  | Point Estimate | 95% Confidence Limits |  |
| Intercept | 3 | -1.147 | 0.964 | -1.190 | 0.253 | -3.201 | 0.907 |  |  |  |
| Intercept | 2 | -0.411 | 0.924 | -0.450 | 0.663 | -2.381 | 1.558 |  |  |  |
| Intercept | 1 | 1.064 | 0.992 | 1.070 | 0.300 | -1.050 | 3.179 |  |  |  |
| Age |  | -0.016 | 0.011 | -1.570 | 0.138 | -0.039 | 0.006 | 0.984 | 0.962 | 1.006 |
| Race/ethnicity | 1 | 0.727 | 0.641 | 1.140 | 0.274 | -0.639 | 2.093 | 2.069 | 0.528 | 8.110 |
| Race/ethnicity | 2 | -1.048 | 1.498 | -0.700 | 0.495 | -4.240 | 2.144 | 0.350 | 0.014 | 8.531 |
| Race/ethnicity | 4 | -0.173 | 0.476 | -0.360 | 0.722 | -1.188 | 0.842 | 0.841 | 0.305 | 2.322 |
| Race/ethnicity | 5 | -0.654 | 1.012 | -0.650 | 0.528 | -2.811 | 1.502 | 0.520 | 0.060 | 4.492 |
| Body mass index | 1 | -0.182 | 1.093 | -0.170 | 0.870 | -2.512 | 2.148 | 0.834 | 0.081 | 8.569 |
| Body mass index | 3 | 0.161 | 0.346 | 0.460 | 0.649 | -0.578 | 0.899 | 1.174 | 0.561 | 2.457 |
| Body mass index | 4 | 0.199 | 0.337 | 0.590 | 0.565 | -0.520 | 0.918 | 1.220 | 0.594 | 2.504 |
| Education | 11 | 0.721 | 0.217 | 3.320 | 0.005 | 0.258 | 1.184 | 2.057 | 1.295 | 3.269 |
| Education | 12 | 0.467 | 0.301 | 1.550 | 0.141 | -0.174 | 1.109 | 1.596 | 0.841 | 3.030 |
| Exercise |  | -0.001 | 0.003 | -0.170 | 0.867 | -0.008 | 0.007 | 0.999 | 0.992 | 1.007 |
| Years since diagnosis |  | 0.009 | 0.011 | 0.780 | 0.446 | -0.015 | 0.032 | 1.009 | 0.985 | 1.032 |
| Lutein and zeaxanthin |  | -0.030 | 0.018 | -1.640 | 0.122 | -0.069 | 0.009 | 0.971 | 0.934 | 1.009 |
|  |  |  |  |  |  |  |  |  |  |  |
| Parameter | | Estimate | Standard Error | t Value | p-value | 95% Confidence Limits |  | Point Estimate | 95% Confidence Limits |  |
| Intercept | 3 | -1.020 | 0.825 | -1.240 | 0.236 | -2.780 | 0.739 |  |  |  |
| Intercept | 2 | -0.290 | 0.780 | -0.370 | 0.715 | -1.953 | 1.373 |  |  |  |
| Intercept | 1 | 1.179 | 0.846 | 1.390 | 0.184 | -0.624 | 2.982 |  |  |  |
| Age |  | -0.022 | 0.011 | -2.010 | 0.063 | -0.045 | 0.001 | 0.978 | 0.956 | 1.001 |
| Race/ethnicity | 1 | 0.510 | 0.597 | 0.860 | 0.406 | -0.761 | 1.782 | 1.666 | 0.467 | 5.942 |
| Race/ethnicity | 2 | -1.267 | 1.717 | -0.740 | 0.472 | -4.926 | 2.392 | 0.282 | 0.007 | 10.930 |
| Race/ethnicity | 4 | -0.260 | 0.476 | -0.550 | 0.593 | -1.275 | 0.755 | 0.771 | 0.279 | 2.128 |
| Race/ethnicity | 5 | -0.517 | 1.046 | -0.490 | 0.628 | -2.746 | 1.712 | 0.596 | 0.064 | 5.540 |
| Body mass index | 1 | -0.504 | 1.182 | -0.430 | 0.676 | -3.024 | 2.015 | 0.604 | 0.049 | 7.504 |
| Body mass index | 3 | 0.315 | 0.361 | 0.870 | 0.397 | -0.454 | 1.083 | 1.370 | 0.635 | 2.954 |
| Body mass index | 4 | 0.399 | 0.299 | 1.330 | 0.202 | -0.239 | 1.037 | 1.491 | 0.788 | 2.822 |
| Education | 11 | 0.745 | 0.210 | 3.550 | 0.003 | 0.298 | 1.192 | 2.106 | 1.347 | 3.294 |
| Education | 12 | 0.488 | 0.273 | 1.790 | 0.094 | -0.094 | 1.070 | 1.629 | 0.911 | 2.914 |
| Exercise |  | -0.001 | 0.003 | -0.270 | 0.792 | -0.008 | 0.006 | 0.999 | 0.992 | 1.006 |
| Years since diagnosis |  | 0.009 | 0.011 | 0.810 | 0.428 | -0.014 | 0.031 | 1.009 | 0.986 | 1.031 |
| trans-Lycopene |  | -0.019 | 0.014 | -1.330 | 0.204 | -0.049 | 0.011 | 0.981 | 0.952 | 1.011 |
|  |  |  |  |  |  |  |  |  |  |  |
| Parameter | | Estimate | Standard Error | t Value | p-value | 95% Confidence Limits |  | Point Estimate | 95% Confidence Limits |  |
| Intercept | 3 | -1.834 | 1.003 | -1.830 | 0.088 | -3.973 | 0.305 |  |  |  |
| Intercept | 2 | -1.142 | 0.980 | -1.160 | 0.262 | -3.231 | 0.948 |  |  |  |
| Intercept | 1 | 0.305 | 1.028 | 0.300 | 0.771 | -1.886 | 2.497 |  |  |  |
| Age |  | -0.011 | 0.012 | -0.940 | 0.364 | -0.037 | 0.015 | 0.978 | 0.956 | 1.001 |
| Race/ethnicity | 1 | 0.712 | 0.599 | 1.190 | 0.253 | -0.565 | 1.990 | 1.666 | 0.467 | 5.942 |
| Race/ethnicity | 2 | -1.003 | 1.502 | -0.670 | 0.515 | -4.205 | 2.199 | 0.282 | 0.007 | 10.930 |
| Race/ethnicity | 4 | -0.304 | 0.475 | -0.640 | 0.533 | -1.317 | 0.709 | 0.771 | 0.279 | 2.128 |
| Race/ethnicity | 5 | -0.343 | 1.055 | -0.320 | 0.750 | -2.592 | 1.907 | 0.596 | 0.064 | 5.540 |
| Body mass index | 1 | -14.501 | 0.678 | -21.390 | <.0001 | -15.946 | -13.056 | 0.604 | 0.049 | 7.504 |
| Body mass index | 3 | 0.282 | 0.341 | 0.830 | 0.421 | -0.445 | 1.009 | 1.370 | 0.635 | 2.954 |
| Body mass index | 4 | 0.345 | 0.311 | 1.110 | 0.284 | -0.317 | 1.008 | 1.491 | 0.788 | 2.822 |
| Education | 11 | 0.681 | 0.207 | 3.290 | 0.005 | 0.240 | 1.122 | 2.106 | 1.347 | 3.294 |
| Education | 12 | 0.488 | 0.276 | 1.770 | 0.098 | -0.101 | 1.077 | 1.629 | 0.911 | 2.914 |
| Exercise |  | 0.000 | 0.003 | -0.040 | 0.970 | -0.007 | 0.006 | 0.999 | 0.992 | 1.006 |
| Years since diagnosis |  | 0.010 | 0.011 | 0.860 | 0.403 | -0.014 | 0.034 | 1.009 | 0.986 | 1.031 |
| Retinyl palmitate |  | -0.074 | 0.074 | -1.000 | 0.332 | -0.233 | 0.084 | 0.981 | 0.952 | 1.011 |
|  |  |  |  |  |  |  |  |  |  |  |
| Parameter | | Estimate | Standard Error | t Value | p-value | 95% Confidence Limits |  | Point Estimate | 95% Confidence Limits |  |
| Intercept | 3 | -1.556 | 0.879 | -1.770 | 0.097 | -3.429 | 0.316 |  |  |  |
| Intercept | 2 | -0.851 | 0.851 | -1.000 | 0.333 | -2.664 | 0.962 |  |  |  |
| Intercept | 1 | 0.615 | 0.914 | 0.670 | 0.511 | -1.332 | 2.562 |  |  |  |
| Age |  | -0.015 | 0.011 | -1.410 | 0.180 | -0.037 | 0.008 | 0.978 | 0.956 | 1.001 |
| Race/ethnicity | 1 | 0.618 | 0.582 | 1.060 | 0.306 | -0.623 | 1.859 | 1.666 | 0.467 | 5.942 |
| Race/ethnicity | 2 | -1.227 | 1.619 | -0.760 | 0.460 | -4.677 | 2.223 | 0.282 | 0.007 | 10.930 |
| Race/ethnicity | 4 | -0.390 | 0.496 | -0.790 | 0.444 | -1.448 | 0.668 | 0.771 | 0.279 | 2.128 |
| Race/ethnicity | 5 | -0.455 | 0.995 | -0.460 | 0.654 | -2.576 | 1.665 | 0.596 | 0.064 | 5.540 |
| Body mass index | 1 | -0.605 | 1.145 | -0.530 | 0.605 | -3.046 | 1.836 | 0.604 | 0.049 | 7.504 |
| Body mass index | 3 | 0.157 | 0.353 | 0.440 | 0.663 | -0.595 | 0.909 | 1.370 | 0.635 | 2.954 |
| Body mass index | 4 | 0.291 | 0.290 | 1.000 | 0.332 | -0.327 | 0.909 | 1.491 | 0.788 | 2.822 |
| Education | 11 | 0.779 | 0.232 | 3.350 | 0.004 | 0.284 | 1.274 | 2.106 | 1.347 | 3.294 |
| Education | 12 | 0.508 | 0.296 | 1.710 | 0.107 | -0.124 | 1.139 | 1.629 | 0.911 | 2.914 |
| Exercise |  | -0.001 | 0.004 | -0.340 | 0.735 | -0.010 | 0.007 | 0.999 | 0.992 | 1.006 |
| Years since diagnosis |  | 0.008 | 0.011 | 0.710 | 0.487 | -0.016 | 0.033 | 1.009 | 0.986 | 1.031 |
| Retinyl stearate |  | -0.239 | 0.250 | -0.960 | 0.353 | -0.771 | 0.293 | 0.981 | 0.952 | 1.011 |
|  |  |  |  |  |  |  |  |  |  |  |
| Parameter | | Estimate | Standard Error | t Value | p-value | 95% Confidence Limits |  | Point Estimate | 95% Confidence Limits |  |
| Intercept | 3 | -1.897 | 0.810 | -2.340 | 0.033 | -3.624 | -0.170 |  |  |  |
| Intercept | 2 | -1.169 | 0.786 | -1.490 | 0.158 | -2.844 | 0.506 |  |  |  |
| Intercept | 1 | 0.294 | 0.844 | 0.350 | 0.732 | -1.505 | 2.093 |  |  |  |
| Age |  | -0.019 | 0.012 | -1.630 | 0.123 | -0.044 | 0.006 | 0.978 | 0.956 | 1.001 |
| Race/ethnicity | 1 | 0.655 | 0.608 | 1.080 | 0.299 | -0.642 | 1.951 | 1.666 | 0.467 | 5.942 |
| Race/ethnicity | 2 | -1.165 | 1.605 | -0.730 | 0.479 | -4.587 | 2.256 | 0.282 | 0.007 | 10.930 |
| Race/ethnicity | 4 | -0.277 | 0.466 | -0.600 | 0.560 | -1.270 | 0.715 | 0.771 | 0.279 | 2.128 |
| Race/ethnicity | 5 | -0.442 | 0.985 | -0.450 | 0.660 | -2.541 | 1.658 | 0.596 | 0.064 | 5.540 |
| Body mass index | 1 | -0.516 | 1.122 | -0.460 | 0.652 | -2.907 | 1.875 | 0.604 | 0.049 | 7.504 |
| Body mass index | 3 | 0.209 | 0.345 | 0.600 | 0.554 | -0.527 | 0.945 | 1.370 | 0.635 | 2.954 |
| Body mass index | 4 | 0.385 | 0.312 | 1.230 | 0.237 | -0.281 | 1.050 | 1.491 | 0.788 | 2.822 |
| Education | 11 | 0.782 | 0.209 | 3.740 | 0.002 | 0.336 | 1.227 | 2.106 | 1.347 | 3.294 |
| Education | 12 | 0.508 | 0.296 | 1.720 | 0.106 | -0.122 | 1.139 | 1.629 | 0.911 | 2.914 |
| Exercise |  | -0.001 | 0.003 | -0.220 | 0.831 | -0.008 | 0.006 | 0.999 | 0.992 | 1.006 |
| Years since diagnosis |  | 0.010 | 0.011 | 0.900 | 0.383 | -0.014 | 0.033 | 1.009 | 0.986 | 1.031 |
| Vitamin A |  | 0.005 | 0.005 | 0.920 | 0.374 | -0.006 | 0.016 | 0.981 | 0.952 | 1.011 |
|  |  |  |  |  |  |  |  |  |  |  |
| Parameter | | Estimate | Standard Error | t Value | p-value | 95% Confidence Limits |  | Point Estimate | 95% Confidence Limits |  |
| Intercept | 3 | -1.817 | 0.794 | -2.290 | 0.037 | -3.509 | -0.124 |  |  |  |
| Intercept | 2 | -1.088 | 0.764 | -1.420 | 0.175 | -2.716 | 0.541 |  |  |  |
| Intercept | 1 | 0.379 | 0.801 | 0.470 | 0.643 | -1.329 | 2.088 |  |  |  |
| Age |  | -0.020 | 0.013 | -1.540 | 0.144 | -0.048 | 0.008 | 0.978 | 0.956 | 1.001 |
| Race/ethnicity | 1 | 0.640 | 0.599 | 1.070 | 0.302 | -0.636 | 1.916 | 1.666 | 0.467 | 5.942 |
| Race/ethnicity | 2 | -1.285 | 1.700 | -0.760 | 0.462 | -4.909 | 2.340 | 0.282 | 0.007 | 10.930 |
| Race/ethnicity | 4 | -0.209 | 0.454 | -0.460 | 0.652 | -1.176 | 0.759 | 0.771 | 0.279 | 2.128 |
| Race/ethnicity | 5 | -0.426 | 0.992 | -0.430 | 0.674 | -2.542 | 1.689 | 0.596 | 0.064 | 5.540 |
| Body mass index | 1 | -0.616 | 1.147 | -0.540 | 0.599 | -3.060 | 1.828 | 0.604 | 0.049 | 7.504 |
| Body mass index | 3 | 0.194 | 0.347 | 0.560 | 0.585 | -0.547 | 0.934 | 1.370 | 0.635 | 2.954 |
| Body mass index | 4 | 0.351 | 0.313 | 1.120 | 0.279 | -0.315 | 1.018 | 1.491 | 0.788 | 2.822 |
| Education | 11 | 0.826 | 0.230 | 3.590 | 0.003 | 0.336 | 1.315 | 2.106 | 1.347 | 3.294 |
| Education | 12 | 0.514 | 0.288 | 1.790 | 0.095 | -0.100 | 1.127 | 1.629 | 0.911 | 2.914 |
| Exercise |  | -0.001 | 0.003 | -0.190 | 0.851 | -0.008 | 0.007 | 0.999 | 0.992 | 1.006 |
| Years since diagnosis |  | 0.010 | 0.011 | 0.880 | 0.392 | -0.014 | 0.033 | 1.009 | 0.986 | 1.031 |
| Vitamin E |  | 0.000 | 0.000 | 0.700 | 0.493 | 0.000 | 0.001 | 0.981 | 0.952 | 1.011 |
|  |  |  |  |  |  |  |  |  |  |  |
| Parameter | | Estimate | Standard Error | t Value | p-value | 95% Confidence Limits |  | Point Estimate | 95% Confidence Limits |  |
| Intercept | 3 | -1.192 | 0.784 | -1.520 | 0.150 | -2.863 | 0.480 |  |  |  |
| Intercept | 2 | -0.462 | 0.749 | -0.620 | 0.547 | -2.059 | 1.135 |  |  |  |
| Intercept | 1 | 1.009 | 0.802 | 1.260 | 0.228 | -0.702 | 2.719 |  |  |  |
| Age |  | -0.021 | 0.011 | -1.910 | 0.076 | -0.043 | 0.002 | 0.978 | 0.956 | 1.001 |
| Race/ethnicity | 1 | 0.530 | 0.590 | 0.900 | 0.383 | -0.728 | 1.788 | 1.666 | 0.467 | 5.942 |
| Race/ethnicity | 2 | -1.245 | 1.677 | -0.740 | 0.469 | -4.820 | 2.329 | 0.282 | 0.007 | 10.930 |
| Race/ethnicity | 4 | -0.256 | 0.479 | -0.530 | 0.601 | -1.277 | 0.765 | 0.771 | 0.279 | 2.128 |
| Race/ethnicity | 5 | -0.583 | 1.007 | -0.580 | 0.571 | -2.730 | 1.564 | 0.596 | 0.064 | 5.540 |
| Body mass index | 1 | -0.540 | 1.162 | -0.460 | 0.649 | -3.017 | 1.938 | 0.604 | 0.049 | 7.504 |
| Body mass index | 3 | 0.316 | 0.357 | 0.890 | 0.390 | -0.445 | 1.077 | 1.370 | 0.635 | 2.954 |
| Body mass index | 4 | 0.379 | 0.301 | 1.260 | 0.227 | -0.263 | 1.021 | 1.491 | 0.788 | 2.822 |
| Education | 11 | 0.723 | 0.212 | 3.410 | 0.004 | 0.271 | 1.176 | 2.106 | 1.347 | 3.294 |
| Education | 12 | 0.504 | 0.265 | 1.910 | 0.076 | -0.060 | 1.068 | 1.629 | 0.911 | 2.914 |
| Exercise |  | -0.001 | 0.003 | -0.310 | 0.761 | -0.008 | 0.006 | 0.999 | 0.992 | 1.006 |
| Years since diagnosis |  | 0.008 | 0.011 | 0.770 | 0.454 | -0.014 | 0.030 | 1.009 | 0.986 | 1.031 |
| Total lycopene |  | -0.007 | 0.008 | -0.930 | 0.366 | -0.024 | 0.009 | 0.981 | 0.952 | 1.011 |
